# Consumption of Discretionary Salt and Salt from Bouillon among Households, Women, and Young Children in Northern Region, Ghana: A Mixed-Methods Study with the Condiment Micronutrient Innovation Trial (CoMIT) Project

**DOI:** 10.1016/j.cdnut.2024.102088

**Published:** 2024-02-06

**Authors:** Jennie N Davis, Sika M Kumordzie, Charles D Arnold, K Ryan Wessells, Kania W Nyaaba, Katherine P Adams, Xiuping (Jenny) Tan, Emily Becher, Stephen A Vosti, Seth Adu-Afarwuah, Reina Engle-Stone

**Affiliations:** 1 University of California, Department of Nutrition, Institute for Global Nutrition, Davis, CA, United States; 2 University of Ghana, Department of Nutrition and Food Science, Legon, Accra, Ghana; 3 University of California, Department of Agriculture and Resource Economics, Institute for Global Nutrition, Davis, CA, United States

**Keywords:** salt, sodium, bouillon, fortification, Ghana, mixed-methods, qualitative, blood pressure, CoMIT

## Abstract

**Background:**

Information on salt consumption patterns is needed to inform the need for and design of salt reduction strategies.

**Objectives:**

In northern Ghana, this study aimed to estimate household consumption of salt, including salt from bouillon, and compare (estimated) women and children’s salt intake to global recommendations; to estimate the proportion of salt consumed from bouillon; and to identify factors, including knowledge, attitudes, and practices, associated with household salt consumption.

**Methods:**

Employing mixed-methods methodology, we conducted a pilot survey (*n* = 369 households enrolled) and focus group discussions (FGDs; *n* = 20) in Tolon and Kumbungu districts (14 urban, 14 rural clusters) (clinicaltrials.gov registry: NCT04632771). Households reported purchases of discretionary salt (DS, “table salt”) and bouillon cubes. DS and total salt (TS; DS+salt from bouillon) consumption for women (15–49 y) and children (2–5 y) were estimated using the Adult Male Equivalent method and compared with global recommendations (<5 g/d women; <3.75 g/d children). Women's salt intake was also predicted from urinary sodium excretion (INTERSALT equation). Associations between DS and TS consumption, as well as household and women's characteristics, were tested with minimally adjusted and multivariable linear mixed-effects models. Qualitative FGD themes were generated using the Framework Method.

**Results:**

From household purchase data, estimated TS consumption exceeded global recommendations for 44% of children [median: 2.9 (IQR: 1.9, 5.2) g/d] and 60% of women [6.0 (4.0, 10.2) g/d]; 35% of children and 50% of women exceeded recommendations from DS alone. Bouillon contributed <25% of households’ TS consumption. Few characteristics were associated with DS or TS consumption. Salient qualitative themes that shaped salt consumption behaviors included salt’s ubiquity as a seasoning, key household members’ influence on food procurement and preparation, and perceptions about health.

**Conclusions:**

Purchase data suggest salt consumption among women and children exceeds recommendations, even when excluding salt from bouillon; food prepared outside the home likely further contributes. Salt reduction interventions may be warranted in this context.

## Introduction

Salt consumption is high worldwide, with adults regularly consuming ≥10 g/d, double the recommended limit of <5 g/d from the WHO and the American Heart Association (AHA) [[1], [2], [3], [4]]. Consuming ≥5 g/d of salt increases risk of developing hypertension and noncommunicable diseases (NCDs), such as cardiovascular disease and stroke [[5], [6], [7]], and salt reduction strategies are recommended to reduce morbidity and mortality associated with NCDs [8,9]. In Ghana, >30% of adults report experiencing hypertension [10], and there has been an increase in cardiovascular disease mortality from 2010 to 2016 [11].

Few studies have assessed salt consumption in Ghana. In studies among adults, salt consumption was ∼6–12 g/d using data from 24-h urinary sodium excretion [[12], [13], [14], [15]] and household salt inventories [16]. Among children (aged 5–12 y), salt consumption was ∼6.4 g/d from 24-h urine data [17]. Despite the variation in these estimates and the challenges of measuring dietary salt [18,19], the studies suggest salt consumption in Ghana exceeds global recommendations. However, as most studies focused primarily on populations aged ≥40 y, with limited data on children, further examination of salt consumption patterns and dietary sources among other populations is needed.

In Ghana, important sources of dietary salt are discretionary salt, including salt added at the table or during cooking, and salt from condiments such as bouillon cubes [20,21]. Salt iodization has been mandatory in Ghana since 1996 [22], and table salt and commonly used condiments such as bouillon cubes and tomato paste are now produced with iodized salt [23,24]. The salt iodization program has reduced iodine deficiency disorders in Ghana [25]; however, iron, vitamin A, and other micronutrient deficiencies persist [26]. Salt and salty condiments such as bouillon have been identified as vehicles for multiple micronutrient fortification (i.e., iodine plus 1 or more additional micronutrients) due to their widespread consumption [24]. To date, there are no national or regional standards in Ghana for fortifying salt and other condiments with additional micronutrients. Thus, the design of micronutrient fortification strategies for salt and salty condiments should consider salt reduction strategies. Moreover, these strategies should be implemented to avoid contradictory salt reduction messages. To help inform nutrition policy discussions related to salt and bouillon (a source of dietary salt) in Ghana, we conducted a mixed-methods study, including a pilot survey and focus group discussions (FGDs), in 2 districts in the Northern Region. Our objectives were to *1*) estimate household consumption of salt, including salt from bouillon, and compare (estimated) women and children’s salt intakes to global recommendations; *2*) estimate the proportion of salt consumed from bouillon; and *3*) identify factors, including knowledge, attitudes, and practices, associated with household salt consumption.

### Methods

This substudy on salt consumption used data from pilot research that included a survey and FGDs (clinicaltrials.gov registry: NCT04632771). The primary objectives of the pilot research were to assess the micronutrient status of adult women and young children and to evaluate household bouillon consumption. The findings informed a planned randomized controlled trial investigating the effects of a multiple micronutrient fortified bouillon cube on the nutrition status of adult women and young children [clinicaltrials.gov registry NCT05178407; CoMIT (Condiment Micronutrient Innovation Trial) Project] [27]. The pilot research was approved by the Ghana Health Services Ethical Review Committee and the Institutional Review Board of the University of California, Davis, and the study was conducted in accordance with the latest Declaration of Helsinki.

### Study design, participants, and sample size

This study was conducted in the Tolon and Kumbungu districts in the Northern Region, Ghana, in 14 urban and 14 rural clusters from October 2020 to March 2021. Our mixed-methods approach employed a convergent parallel research design, such that quantitative and qualitative collection methods were developed and implemented in parallel [28]. The primary method of data collection was a quantitative cross-sectional pilot survey that recruited nonpregnant, nonlactating women of reproductive age (WRA: 15–49 y), nonpregnant lactating women (LW) aged 15–49 y and 4–18 mo postpartum, and children aged 2–5 y. Qualitative data were collected through FGDs that recruited women aged 15–49 y, adult men aged ≥18 y, and women aged >49 y who had knowledge of or made decisions about household food procurement and cooking practices. Potential participants were excluded if they suffered from any illnesses that precluded study participation, such as congenital anomalies or malignancies, or failed the COVID-19 screening [fever and/or recent (within 14 d) exposure to COVID-19]. All participants provided written informed consent. Additional study design and recruitment details can be found in **Supplemental Methods**.

From the pilot survey, we included 487 women (WRA+LW) in our analysis. With this sample size, we were able to detect a strength of correlation in bivariate associations with household salt consumption of ≥0.14 (80% power, α = 0.05). For the FGDs, we aimed to recruit a convenience sample of 120 participants (*n* = 60; women aged 15–49 y, *n* = 30 men, *n* = 30 women aged >49 y).

### Data collection procedures—quantitative

For the pilot survey, trained fieldworkers recruited households and administered questionnaires in the household’s preferred language (English or Dagbani, the primary local dialect) using electronic tablets programmed with SurveyCTO software. Household heads or other knowledgeable household members completed a household questionnaire that collected data on the following: demographics, water and sanitation [29,30], assets, food insecurity status [31], and household purchases of fortified foods (e.g., bouillon, salt) using the Fortification Assessment Coverage Toolkit (FACT) [32]. Participating WRA and LW within recruited households then completed a modified WHO STEPwise approach to NCD risk factor surveillance (STEPS, version 3.2) questionnaire [33] (modified to include questions on the consumption frequency of salty snacks, such as salty chips/crisps or fried foods), and a Knowledge, Attitudes, and Practices (KAP) questionnaire developed for the pilot survey to collect information on consumption of bouillon, salt, and “dawadawa” (a condiment made from fermented locust beans that flavors many common local dishes [34]), and included questions from the Marlowe–Crowne Social Desirability Scale [35]. Participants visited a biospecimen collection site central to each cluster after the household visit. Among WRA and children only, measurements of standing height, weight, and blood pressure were recorded (all measurements were conducted in triplicate); WRA also provided a one-spot urine sample to measure urinary sodium, potassium, and creatinine. Additional methodological details and definitions can be found in Supplemental Methods.

### Data collection procedures—qualitative

The FGDs (*n* = 20) were held in community spaces central to each cluster, such as schools or health centers, and participants completed a demographic questionnaire and COVID-19 screening. Trained facilitators (*n* = 3) conducted the FGDs in Dagbani following a semistructured FGD guide (**Supplemental Appendix**) focused primarily on the purchase and consumption of bouillon, with a subset of questions on salt consumption behaviors and relationships between salt and health. The FGD guide was developed, translated, piloted, and revised with in-country collaborators and fieldworkers, considering the study setting, context, and culture. FGDs were conducted between groups of 5–6 participants (women aged 15–49 y, *n* = 10 FGDs; men aged ≥18 y, *n* = 5 FGDs; women aged >49 y, *n* = 5 FGDs), and included a note-taker who systematically gathered participant responses and reactions during the FGDs with a written form. The FGDs were audio-recorded and lasted ∼2 h, followed by a debriefing with the facilitator, note-taker, and supervisor using a written debriefing form.

### Data analysis

Statistical analysis plans were posted publicly before analysis (https://osf.io/t3zrn/). For this mixed-methods study, quantitative and qualitative analyses were performed separately, with qualitative data used to understand and interpret quantitative findings [36]. Quantitative analyses were completed using Stata (16.1, StataCorp LLC, College Station) and qualitative analyses with NVivo (QSR International Pty Ltd., released March 2020).

#### Quantitative analyses

Household food insecurity status was calculated using the Household Food Insecurity Access Scale [31]. BMI (WRA) categories were defined according to standard categories [37]; anthropometric *z*-scores (children) were calculated according to WHO child growth standards [38].

For WRA, 3 definitions of elevated blood pressure and hypertension are presented to capture a broader picture of hypertension risk: AHA [39], the International Society of Hypertension (ISH) [40], and WHO [41] (Supplemental Table 1). For children, “at risk” blood pressure thresholds were defined by the American Academy of Pediatrics [42] (Supplemental Table 2).

Urinary sodium and potassium concentrations were determined by inductively coupled plasma spectrometry, and urine creatinine was measured with the Cayman creatinine colorimetric assay kit (Supplemental Methods) [43]. Sodium-to-potassium ratios were calculated by dividing mean urinary sodium (mmol/L) by mean urinary potassium (mmol/L); a ratio of ≤1 indicated a diet potentially lower in sodium or protective against hypertension, though the evidence is still insufficient to conclusively determine these correlations [44].

#### Estimating household and individual salt consumption from purchase data

Estimated median (IQR) daily consumption of household discretionary salt (table salt) and total salt (discretionary salt plus salt from bouillon) were calculated from purchase data collected with the FACT questionnaire, which defines the purchase recall period as “since the last time” the food item was acquired [32]. Though other contributors to total dietary salt exist, such as processed foods or restaurant meals, we were not able to assess these contributions from the available data; thus, for brevity, we henceforth refer to the combination of discretionary salt and salt from bouillon as “total salt.” Household daily discretionary salt consumption (g/d) was estimated by dividing the quantity of salt last purchased in grams by the total number of days that quantity was reported to typically last [45,46]. This calculation was repeated for bouillon, with the total multiplied by 55% (bouillon estimated to contain 55% salt) [24] to estimate salt consumption from bouillon. Before estimating household daily discretionary and total salt consumption, the variables’ distributions were examined and all observations were retained. Estimated median (IQR) daily consumption of discretionary and total salt of individuals (WRA and children) were calculated using the Adult Male Equivalent (AME) method in which household level daily consumption of discretionary and total salt was assumed to be distributed within the household in proportion to each household member’s age- and sex-specific energy requirements [45,46] (Supplemental Methods). Reporting of estimated individual consumption with the AME method is often referred to as “apparent intake” or “apparent consumption” to reflect this assumption [45]; hereafter, it is referred to as “consumption.”

We then examined estimates of individual salt consumption in relation to international recommendations. We defined high salt consumption for WRA as ≥5 g/d [8]. We defined high salt consumption for children aged 2–3 y as ≥3 g/d and ≥3.75 g/d for children aged 4–5 y, according to the National Academies of Sciences 2019 Chronic Disease Risk Reduction recommendations [44].

#### Estimating individual salt consumption from spot urine samples

Daily total salt consumption was estimated from spot urine samples from WRA. We used the INTERSALT equation to predict 24-h urinary sodium excretion [47] and then estimated daily total salt consumption (g/d) by dividing the predicted 24-h urinary sodium excretion (mg/d) by 390 as there are 390 mg sodium in 1 g sodium chloride (table salt) [15] (Supplemental Methods). Estimates were then compared with international salt recommendations. The INTERSALT equation was selected as it has separate equations for males and females and has been evaluated in populations of African descent [[47], [48], [49]]. Use of spot urine samples to predict 24-h sodium excretion (and subsequent daily salt consumption) is recommended only for estimations of population salt consumption above or below a threshold, such as <5 g/d [50]. Use of this method to estimate individual salt consumption or predict clinical outcomes, such as hypertension, is not recommended due to within-person variability in urinary sodium excretion, measurement error in urine collection, and bias built into the predictive equations [48,50,51].

#### Identifying factors associated with estimated household salt consumption from purchase data

Using linear mixed-effects models, we examined factors associated with estimated household salt consumption (total and discretionary) from the purchase data, where district, setting (urban compared with rural), household size, and participant type (WRA or LW) were included as separate fixed effects, and cluster as the random effect. Factors examined were selected based on theoretical potential relationships with salt consumption. These included household and individual characteristics (e.g., household asset index, household food insecurity, education level, individual blood pressure measurements) and factors related to KAP [6,20,52,53]. Models included factors at both household and individual levels (WRA+LW) as the perceptions of women may have influenced their households’ salt consumption. All factors were selected *a priori* according to our analysis plan (Supplemental Table 3).

Outcome variables were 1) estimated household discretionary salt consumption and 2) estimated household total salt consumption (continuous, g/d). Before model analysis and to meet regression assumptions, we identified values that were less than the 2.5 percentile (*n* = 8, 1.7% discretionary salt; *n* = 9, 1.9% total salt) or greater than the 97.5 percentile (*n* = 9, 1.9% discretionary salt; *n* = 9, 1.9% total salt) and replaced them with the value at the respective percentile thresholds (Supplemental Figure 1); sensitivity analyses were conducted with all values included (i.e., before replacement). For the linear mixed-effects analysis, all continuous factors (e.g., household size, age) were categorized by either dividing the data into commonly used categories (i.e., age categories of 15–24 y, 25–34 y, 25–49 y) or following natural breaks in the factor’s distribution. For the analysis, each categorical factor listed in Supplemental Table 3 was first tested with each outcome variable in separate minimally adjusted, mixed-effects models. Then, marginally statistically significant factors (*P* < 0.1) were included in a multivariable mixed-effects model. Collinearity between factors was assessed with variance inflation factors (>5) and tolerance (<0.1). To inform model interpretation, we constructed a correlation matrix among all factors, including variables of social desirability (Supplemental Figure 2).

Exploratory analyses were conducted by constructing additional linear mixed-effects models to test associations between groups of KAP-related factors and estimated household discretionary and total salt consumption. Analysis methods can be found in Supplemental Methods.

#### Qualitative analyses

FGD audio recordings were translated and transcribed to English by a fieldworker not involved with the FGDs. A random subset of the audio recordings was translated and transcribed by a second independent transcriptionist (*n* = 7 total: *n* = 3 women aged 15–49 y, *n* = 2 men, *n* = 2 women aged >49 y from rural and urban clusters), with discrepancies resolved through discussion.

A codebook was developed based on the FGD guide, notes, and debriefing forms and followed a format of segments, structural codes, and content codes [[54], [55], [56]]. Segments, defined as the primary questions from the FGD guide, were subdivided into structural codes, defined as the subquestions and all relevant probes within each primary question. Multiple content codes were then applied to each structural code. The initial list of content codes was updated and refined throughout the coding process through discussions between the 2 coders (SMK, cultural insider [emic]; JND, cultural outsider [etic]) [57,58].

Using the codebook, JND and SMK independently coded FGD transcripts (*n* = 7, 35%) applying a line-by-line directed content coding strategy, meaning that each line of text or phrase could be applied to multiple content codes [55]. Double-coded transcripts were compared, and consistency was assessed with intercoder reliability (ICR) score (Cohen’s Kappa) and percent agreement. ICR was calculated for each transcript segment [54,59]. Segments with an ICR score of <0.7 (ICR ≥ 0.7 indicates substantial agreement) [59] were discussed, independently recoded, and the ICR score recalculated. The average final ICR score was 0.95, and the percent agreement was 96.5% (Supplemental Table 4). Double-coded and revised transcripts were uploaded into NVivo, and the remaining transcripts (*n* = 13) were coded in NVivo directly by JND.

Content analysis and thematic selection followed the Framework Method [58]. Framework matrixes were generated with NVivo, wherein the coded data were reduced and summarized in data matrixes. JND, SMK, and RES reviewed the data matrixes and discussed salient themes. Final themes were agreed upon after triangulation with data from note-taking and debriefing forms and discussions with fieldworkers.

## Results

### Characteristics of participating households, women, and children in the pilot survey

Fieldworkers visited 375 households, 371 provided consent (99%), and 369 participated in the pilot survey (98%) (Supplemental Figure 3). Demographic and socioeconomic characteristics of the households are presented in Table 1. Notably, 77% of households in both urban and rural strata were moderately to severely food insecure. Among WRA and LW (*n* = 487), most were married, had not completed primary school, and were self-employed. Among WRA only, underweight affected 9%, and 20% had overweight or obesity; hypertension affected 17% (AHA definition) and 6% (ISH/WHO definition). Less than 1% (*n* = 1) had a sodium-to-potassium ratio of <1 (Table 2). Among children, wasting and stunting affected 5% and 32%, respectively. The children with blood pressure measurements considered at risk were 19% males and 12% females (Table 2).

**TABLE 1:** Characteristics of households, women, and children who participated in the pilot survey in Tolon and Kumbungu districts, northern Ghana: CoMIT project^1^

Characteristics	**Overall**	**Urban**	**Rural**
	*n* (%)	*n* (%)	*n* (%)
Household			
Total households	369 (100)	172 (47)	197 (53)
Household size (median [IQR])	10 (8,14)	11 (8,14)	10 (7,14)
Education level of household head			
None	100 (27)	22 (13)	78 (39)
Primary school	56 (15)	21 (12)	35 (18)
Secondary or higher	213 (58)	129 (75)	84 (43)
Has electricity	308 (84)	172 (100)	136 (70)
Access to an improved water source	203 (55)	134 (78)	69 (35)
Access to an improved toilet	108 (29)	80 (47)	28 (14)
Food insecurity status			
Food secure	20 (5)	18 (10)	2 (1)
Mild insecurity	66 (18)	24 (14)	42 (21)
Moderate insecurity	193 (52)	86 (50)	107 (54)
Severe insecurity	90 (25)	44 (26)	46 (24)
Women			
Total	487 (100)	238 (49)	249 (51)
Age (y), median (IQR)	30 (24, 35)	30 (23, 35)	30 (24, 36)
Highest education level completed			
None	345 (71)	145 (61)	200 (80)
Primary school	55 (11)	30 (13)	25 (10)
Secondary or higher	86 (18)	62 (26)	24 (10)
Marital status			
Never married	56 (11)	31 (13)	25 (10)
Currently married	416 (86)	198 (84)	218 (88)
Widowed or separated	13 (3)	8 (3)	5 (2)
Employment status			
Self-employed	337 (70)	170 (72)	167 (67)
Homemaker	79 (16)	33 (14)	46 (18)
Student	34 (7)	20 (8)	14 (6)
Other	35 (7)	14 (6)	21 (9)
Children			
Total	246 (100)	123 (50)	123 (50)
Age (y), median (IQR)	3 (2, 4)	3 (2, 4)	3 (2,4)
Female	116 (47)	53 (43)	63 (51)

Abbreviations: CoMIT, Condiment Micronutrient Innovation Trial.

1 Women include nonpregnant, nonlactating women of reproductive age (15–49 y; *n* = 244), and nonpregnant lactating women (15–49 y, 4–18 mo postpartum; *n* = 243). Children were aged 2–5 y. Improved water sources and improved toilets were defined according to the WHO/UNICEF Joint Monitoring Programme [29,30]. Food insecurity was calculated using the Household Food Insecurity Access Scale score [31]. “Other” employment status includes those who were nonpaid (*n* = 1), government employees (*n* = 3), or unemployed but able to work (*n* = 31).

**TABLE 2:** Biological characteristics for nonpregnant, nonlactating women of reproductive age (15–49 y), and children (2–5 y) who participated in the pilot survey in Tolon and Kumbungu districts, northern Ghana: CoMIT Project^1^

Characteristics	***n***	**Overall**	***n***	**Urban**	***n***	**Rural**
Women						
BMI, kg/m^2^	222	22.4 ± 3.9	106	22.8 ± 4.5	116	22.1 ± 3.1
BMI category,^2^*n* (%)						
Underweight		21 (9)		13 (12)		8 (7)
Normal weight		157 (71)		69 (65)		88 (76)
Overweight		34 (15)		17 (16)		17 (15)
Obese		10 (5)		7 (7)		3 (3)
Blood pressure, mmHg^3^						
Systolic	223	110.8 ± 16.1	106	110.8 ± 15.3	117	110.9 ± 16.9
Diastolic	223	69.8 ± 10.8	106	69.7 ± 9.3	117	69.8 ± 12.1
AHA elevated blood pressure,^3^*n* (%)		13 (6)		7(7)		6 (5)
AHA hypertension,^3^*n* (%)		38 (17)		16 (15)		22 (19)
ISH high-normal blood pressure,^3^*n* (%)		14 (6)		9 (9)		5 (4)
ISH and WHO hypertension,^3^*n* (%)		13 (6)		5 (5)		8 (7)
Urinary sodium excretion,^4^ mmol/L	219	142.9 ± 72.4	103	135.8 ± 72.1	116	149.2 ± 72.3
Urinary potassium excretion,^4^ mmol/L	219	35.0 ± 26.2	103	33.7 ± 26.2	116	36.2 ± 26.2
Urinary creatinine excretion,^4^ mg/dL	220	130.0 ± 84.6	104	133.2 ± 92.1	116	127.2 ± 77.6
Sodium-to-potassium ratio	219	5.7 ± 4.0	103	5.7 ± 4.1	116	5.6 ± 3.9
Predicted urinary sodium excretion,^4^ mg/d	219	2756.4 ± 558.2	103	2795.5 ± 547.9	116	2712.3 ± 569.1
Children						
BAZ	232	−0.4 ± 1.0	114	−0.5 ± 1.0	118	−0.4 ± 1.0
BAZ category,^2^*n* (%)						
Underweight		9 (4)		3 (3)		6 (5)
Normal		219 (94)		109 (96)		110 (93)
At risk of being overweight		4 (2)		2 (2)		2 (2)
Stunted, 2*n* (%)		73 (32)		27 (24)		46 (39)
Wasted, 2*n* (%)		11 (5)		3 (3)		8 (7)
Blood pressure, mmHg						
Systolic	184	90.3 ± 10.8	96	90.7 ± 10.2	88	89.9 ± 11.5
Diastolic	184	55.0 ± 6.7	96	55.6 ± 6.6	88	54.3 ± 6.8
Blood pressure “at risk,” 3*n* (%)						
Female		22 (12)		11 (12)		11 (13)
Male		35 (19)		21 (22)		14 (16)

Abbreviations: AHA, American Heart Association; APA, American Pediatric Association; BAZ, BMI-for-age *z*-score; CoMIT, Condiment Micronutrient Innovation Trial; ISH, International Society of Hypertension.

1 Results are presented as mean ± SD unless otherwise indicated.

2 Women BMI categories: underweight, BMI < 18.5 kg/m^2^; normal weight, BMI ≥ 18.5–24.9 kg/m^2^; overweight, BMI 24.0–25.9 kg/m^2^; obese BMI ≥ 30.0 kg/m^2^. Child BAZ categories: underweight, BAZ <−2 SD; normal weight, BAZ −2 ≥ SD ≤ 2; and at risk of overweight, BAZ >2 SD. Stunted, Height-for-age *z*-score <−2 SD; wasted, weight-for-height *z*-score <−2 SD.

3 Blood pressure measurements for women and children are the average of 3 measurements taken in the same sitting using an automatic portable upper-arm blood pressure monitor. Blood pressure guidelines and thresholds: AHA adult elevated blood pressure: systolic 120–129 and diastolic <80 mmHg; AHA adult hypertension: systolic ≥ 130 or diastolic ≥ 80 mmHg [39]. ISH high-normal blood pressure: systolic 130–139 and/or diastolic 85–89 mmHg; ISH and WHO hypertension: systolic ≥ 140 and/or diastolic ≥ 90 mmHg [40,41]. “At risk” blood pressure for children is listed in Supplemental Table 2 [42].

4 Analysis of urinary sodium, potassium, and creatinine completed from a single spot urine sample from women. Predicted urinary sodium excretion (g/d) was calculated using the INTERSALT formula [47].

### Estimated salt consumption among women, children, and households

**FIGURE 1:**
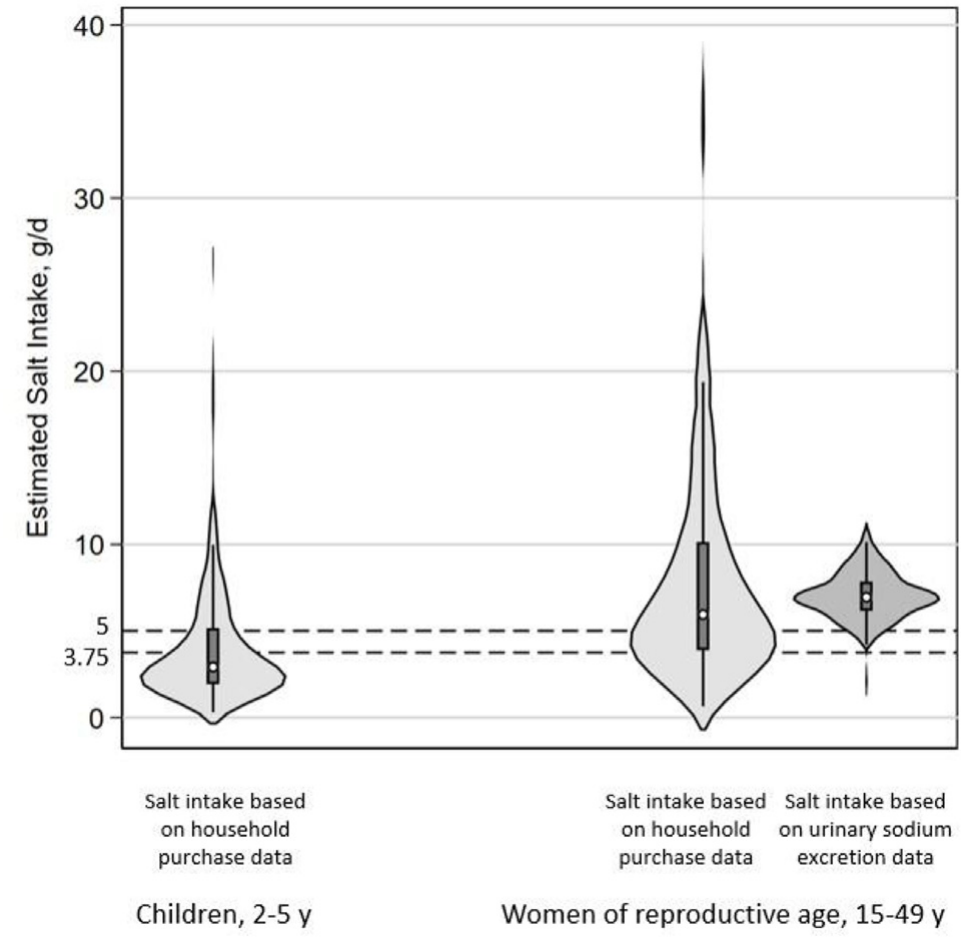
Violin plots of estimated salt consumption distributions among women and children participating in the pilot survey in the Tolon and Kumbungu districts, northern Ghana: CoMIT project. Darkened boxes within each violin plot represent the interquartile range of the data and circles represent the median. Salt intake based on household purchase data indicates estimated total salt consumption (discretionary salt + salt from bouillon) disaggregated from household purchase data collected using the Fortification Assessment Coverage Toolkit (*n* = 237 women; *n* = 239 children) [32]. Salt intake based on urinary sodium excretion (women only, *n* = 219) indicates salt intake calculated from urinary sodium excretion data using the INTERSALT prediction equation [47]. Dashed lines represent the global recommended threshold to consume <5/g of salt for adults [8], and <3.75 g/d for children [44]. CoMIT, Condiment Micronutrient Innovation Trial.

Estimates of discretionary and total salt consumption for individuals and households and predicted total salt intake calculated from urinary sodium excretion are presented in Table 3. The majority of WRA (60%, *n* = 141) and 44% of children (*n* = 105 total: *n* = 70 children aged 2–3 y, *n* = 35 children aged 4–5 y) appeared to exceed global recommendations for daily total salt consumption; 35% of children and 50% of women seemed to exceed recommendations from discretionary salt alone (Figure 1). Median total salt intake was ∼25% more in rural areas than in urban areas among women, children, and households. Bouillon comprised ∼23% of household total salt consumption.

**TABLE 3:** Apparent intake of salt of nonpregnant, nonlactating women (15–49 y), and children (2–5 y), and estimated salt consumption of households, based on household estimates or urinary sodium excretion among those who participated in the pilot survey in the Tolon and Kumbungu districts, northern Ghana: CoMIT Project^1^

		Estimated discretionary salt consumption from purchase data (g/d)		Estimated total salt consumption from purchase data (g/d)	Proportion of total salt consumption from bouillon		Predicted total salt intake from urinary sodium excretion (g/d)	
	*n*	Median (IQR)	*n*	Median (IQR)	%	*n*	Median (IQR)	Mean (SD)
Women (15–49 y)	239	5.1 (3.1, 8.9)	237	6.0 (4.0, 10.2)	14.3%	219	7.1 (6.2, 7.9)	7.1 (1.4)
Urban	116	4.3 (2.8, 7.3)	115	5.4 (3.6, 8.9)	20.8%	103	7.0 (6.0, 7.8)	7.0 (1.5)
Rural	123	6.3 (3.6, 10.2)	122	7.1 (4.2, 11.5)	11.2%	116	7.2 (6.3, 8.1)	7.2 (1.4)
Children (2–5 y)	241	2.4 (1.6, 4.2)	239	2.9 (1.9, 5.2)	16.7%	--	--	--
Urban	120	2.2 (1.4, 3.8)	119	2.6 (1.3, 4.2)	17.0%	--	--	--
Rural	121	2.8 (1.7, 4.8)	120	3.3 (2.1, 5.4)	14.7%	--	--	--
Households	363	43.1 (32.1, 106.9)	360	56.2 (40.3, 116.2)	23.2%	--	--	--
Urban	168	41.0 (28.6, 85.7)	167	52.3 (39.2, 102.2)	21.7%	--	--	--
Rural	195	55.0 (33.3, 106.9)	193	66.5 (41.2, 117.9)	17.3%	--	--	--

Abbreviation: CoMIT, Condiment Micronutrient Innovation Trial.

1 Estimated discretionary salt and total salt (discretionary salt + salt from bouillon, where bouillon was assumed to be 55% salt) were calculated from purchase data using the Fortification Assessment Coverage Toolkit [32]. Estimations for women and children are presented as grams per day per Adult Male Equivalent. Predicted urinary sodium excretion (mg/d) was estimated using the INTERSALT equation [47], and then predicted total salt intake (g/d) was calculated by dividing the estimate of urinary sodium excretion (g/d) by 390 as there is 390 mg sodium in 1 g sodium chloride (table salt) [15].

### Factors associated with daily household salt consumption

Factors associated with daily household discretionary and total salt consumption were similar in minimally adjusted and multivariable models **[**Table 4 **(total salt);** Supplemental Table 5 **(discretionary salt);** Supplemental Figure 4**]**. In the total salt consumption multivariable model, household factors of size (number of household members; *P* < 0.001) and food insecurity (*P* = 0.010) were positively associated with household total salt consumption. The attitude that it is “very important” to lower dietary salt (compared with “somewhat important”) and the practice of doing ≥1 action regularly to control dietary salt (compared with no regular actions) were negatively associated with household total salt consumption. The attitude that it is not important to lower dietary salt (compared with “somewhat important”) was positively associated with household total salt consumption. No other associations were found with other household or individual demographics, KAP, or dietary factors in the total salt consumption multivariable model. However, household assets were marginally associated with total and discretionary salt consumption in minimally adjusted models (*P* = 0.073 and *P* = 0.060, respectively). Results from sensitivity analyses with all values (i.e., no replacement of values below the 2.5 and above the 97.5 percentiles) were similar to those described above, as were the results from exploratory analyses of the effect of groups of KAP predictors on daily household discretionary and total salt consumption (Supplemental Table 6).

**TABLE 4:** Factors associated with household daily total salt consumption among households who participated in the pilot survey in Tolon and Kumbungu districts, northern Ghana: CoMIT Project^1^

Variable	Category	Household total salt consumption, g/d		Minimally adjusted analyses^2^		Multivariable analyses^2^	
		*n*	Mean (SD)	*β* (95% CI)	*P*	*β* (95% CI)	*P*
Household level factors							
Size, No. of members	1–8	117	62.5 (54.3)	ref.	<0.001	ref.	<0.001
	9–11	131	71.4 (47.3)	7.9 (−6.2, 22.0)		0.9 (−13.4, 15.2)	
	12–15	112	85.8 (57.5)	27.1 (12.0, 42.1)		9.1 (−7.7, 25.8)	
	16+	112	117.8 (70.2)	53.9 (38.9, 68.8)		37.4 (19.7, 55.0)	
District	Tolon	238	87.3 (66.3)	ref.	0.118	ref.	0.675
	Kumbungu	234	79.9 (54.9)	−9.8 (−22.1, 2.5)		2.9 (−10.8, 16.7)	
Setting	Urban	230	76.1 (55.5)	ref.	0.010	ref.	0.094
	Rural	242	90.8 (65.1)	16.2 (3.9, 28.5)		11.6 (−2.0, 25.1)	
Participant type	WRA	237	82.3 (59.9)	ref.	0.728	ref.	0.258
	Lactating women	235	62.1 (84.9)	1.8 (−8.3, 11.9)		6.4 (−4.7, 17.5)	
Asset quintiles	First (lowest)	87	62.5 (47.5)	ref.	0.073	ref.	0.356
	Second	81	85.2 (63.4)	16.3 (−1.1, 33.6)		17.0 (−0.7, 34.7)	
	Third	95	96.7 (66.9)	23.2 (6.1, 40.3)		14.9 (−2.6, 32.5)	
	Fourth	107	85.5 (59.9)	7.7 (−9.8, 25.2)		12.7 (−6.1, 31.4)	
	Fifth (highest)	102	86.1 (61.1)	14.2 (−4.7, 33.1)		16.1 (−5.3, 37.4)	
Food insecurity	None to mild	113	74.7 (50.4)	ref.	<0.001	ref.	0.010
	Moderate	247	73.5 (53.2)	3.4 (−8.9, 15.8)		−3.9 (−18.7, 11.0)	
	Severe	114	114.2 (74.8)	38.1 (24.0, 52.3)		28.3 (8.3, 48.3)	
Hh head education	None	109	81.2 (64.9)	ref.	0.451		
	Preschool or primary	71	92.3 (66.8)	10.1 (−7.1, 27.3)			
	Secondary or greater	282	82.4 (57.9)	1.5 (−12.1, 15.2)			
Dawadawa consumption	Low	104	73.9 (44.7)	ref.	0.587		
	Medium	282	88.4 (65.1)	5.6 (−7.6, 18.8)			
	High	57	72.0 (65.0)	−1.1 (−19.7, 17.5)			
Demographic factors (individual level)							
Women age, y	15–24 y	126	82.9 (63.8)	ref.	0.975		
	25–34 y	204	86.6 (63.1)	−1.4 (−14.2, 11.)			
	35–49 y	138	81.0 (55.4)	−0.3 (−14.0, 13.5)			
History of heart disease	No	434	82.4 (60.5)	ref.	0.499		
	Yes	36	100.3 (65.8)	6.7 (−12.7, 26.0)			
Current hypertension	No	202	83.8 (62.6)	ref.	0.577		
	Yes	12	81.8 (42.2)	9.5 (−23.9, 42.8)			
BMI	Normal	153	87.8 (62.0)	ref.	0.346		
	Underweight	20	81.9 (58.9)	−0. −26.9, 25.6)			
	Overweight/obese	43	69.6 (59.6)	−14.0 (−33.0, 5.0)			
Dietary patterns (individual level)							
Vegetable consumption	1–2 d/wk	56	61.6 (53.0)	ref.	<0.001	ref.	0.438
	3–5 d/wk	213	72.2 (53.9)	9.3 (−6.7, 25.4)		6.1 (−13.4, 25.7)	
	6–7 d/wk	201	102.2 (65.2)	40.7 (23.6, 57.9)		14.3 (−8.7, 37.3)	
Fruit consumption	None	125	66.1 (50.9)	ref.	0.001	ref.	0.244
	1–2 d/wk	282	85.2 (61.8)	14.1 (2.2, 25.9)		1.4 (−12.8, 15.6)	
	≥3 d/wk	63	112.7 (64.8)	33.7 (16.1, 51.2)		16.3 (−5.1, 37.7)	
Salty snacks consumption	None	161	77.6 (52.1)	ref.	0.967		
	1–2 d/wk	224	85.7 (67.8)	−1.9 (−16.6, 12.8)			
	≥3 d/wk	85	90.6 (57.2)	−1.7 (−18.7, 15.3)			
Consumption of foods made with bouillon	0–6 d/wk	24	51.3 (28.0)	ref.	0.083	ref.	0.248
	7 d/wk	311	82.0 (60.0)	20.3 (−2.7, 43.3)		12.7 (−8.9, 34.3)	
Knowledge factors (individual level)							
Quantity of salt consumed	Just the right amount	363	84.2 (59.0)	ref.	0.923		
	Too much	25	88.3 (53.0)	2.0 (−21.0, 24.9)			
	Too little	82	80.7 (71.6)	2.6 (−11.0, 16.3)			
Think dietary salt causes health problems	No	42	62.6 (43.4)	ref.	0.060	ref.	0.301
	Yes	374	87.8 (62.3)	22.0 (3.7, 40.3)		16.9 (−4.9, 38.8)	
	Do not know	54	72.8 (59.7)	17.1 (−5.7, 39.8)		17.5 (−9.2, 44.1)	
Attitude factors (individual level)							
Importance of lowering salt in the diet	Somewhat important	168	80.4 (59.4)	ref.	<0.001	ref.	0.013
	Very important	187	67.2 (47.7)	−13.7 (−25.1, -2.3)		−2.8 (−16.8, 11.2)	
	Not at all important	115	115.8 (70.3)	26.3 (13.2, 39.5)		24.4 (6.6, 42.1)	
Perception of dietary salt	No effect	91	75.3 (44.5)	ref.	0.319		
	Good	141	88.9 (64.8)	6.1 (−8.6, 20.7)			
	Bad	103	71.3 (60.1)	−4.9 (−20.4, 10.5)			
Perception of dietary dawadawa	No effect	32	75.8 (51.0)	ref.	0.975		
	Good	303	80.2 (59.7)	0.3 (−20.7, 21.4)			
	Bad	0	--	--			
Perception of dietary bouillon	No effect	48	87.8 (57.9)	ref.	0.123		
	Good	231	77.3 (60.4)	−16.0 (−33.2, 1.2)			
	Bad	56	83.3 (53.0)	−5.6 (−26.7, 15.6)			
Practice factors (individual level)							
Add salt at the table	Always/often/sometimes	194	96.2 (62.2)	ref.	0.011	ref.	0.429
	Rarely/never	276	75.0 (58.7)	−13.7 (−24.2, -3.2)		−4.9 (−17.2, 7.3)	
Add salt during cooking	Always/often/sometimes	449	83.5 (61.8)	ref.	0.453		
	Rarely/never	21	90.0 (42.6)	9.5 (−15.3, 34.2)			
Eat processed foods	Always/often/sometimes	232	79.8 (63.6)	ref.	0.258		
	Rarely/never	238	87.8 (58.3)	6.0 (−4.4, 16.5)			
Do ≥1 action regularly to control dietary salt	No	91	86.1 (49.9)	ref.	0.068	ref.	0.024
	Yes	379	83.2 (63.4)	−12.1 (−25.1, 0.9)		−18.5 (−34.5, −2.5)	

1 “Total salt” is defined as discretionary salt (table salt) plus the proportion of salt from bouillon, where bouillon was assumed to be 55% salt [24]. Estimates of Hh total salt consumption were calculated from purchase data using the Fortification Assessment Coverage Toolkit [32]. Hh total salt consumption (g/d) was presented as an unadjusted mean (SD)—households with total salt data: *n* = 360. Hh head education is defined as the highest education level completed. Hh consumption of dawadawa categories defined as: low, 0–10 g/d; med, 10.01–48.9 g/d; high, >49 g/d, where the cutoffs for each category were determined by the natural cutoffs present in the variable’s distribution. History of heart disease was self-reported. Hypertension is defined according to the WHO definition: systolic blood pressure ≥ 140 mmHg and/or diastolic blood pressure ≥ 90 mm Hg [41]. BMI categories defined as normal, 18.5–24.9 kg/m^2^; underweight, <18.5 kg/m^2^; overweight/obese, ≥25.0 kg/m^2^. Salty snacks are defined as salty crisps, chips, nuts, or salty fried foods eaten between main meals. CoMIT, Condiment Micronutrient Innovation Trial; Hh, household; ref., reference group.

2 Minimally adjusted and multivariable analyses were linear mixed-effects regression models that controlled for Hh size, district (Tolon/Kumbungu), area (urban/rural), and participant type (woman of reproductive age or lactating woman) as fixed effects and cluster as a random effect. The outcome variable (Hh total salt consumption, g/d) was tested as a continuous variable, and all predictors were categorical variables. Predictors were included from both the household level and individual level (from data from nonpregnant, nonlactating women of reproductive age, 15–49 y, and nonpregnant lactating women, 6–18 mo postpartum, aged 15–49 y; *n* = 487 total). After being tested separately in minimally adjusted models, marginally significant predictors (*P* < 0.1, **bolded**) were included in the multivariable model.

In correlation analyses with social desirability variables (Supplemental Figure 2), the practices of adding salt at the table and doing ≥1 action regularly to control salt intake were strongly correlated with social desirability variables (correlation ranged from 0.5 to 0.6 and 0.8 to 0.9, respectively). The attitude of believing in the benefits of salt consumption and weekly vegetable consumption were moderately correlated with social desirability variables (correlation ranged from 0.3 to 0.5 and 0.2 to 0.5, respectively).

We conducted post hoc analyses to better understand the associations between household asset scores, food insecurity, and salt consumption. We found that discretionary salt consumption increased with greater food insecurity status within each asset quintile except the third quintile, and households in the lowest (first and second) asset quintiles last purchased smaller quantities of salt than other quintiles (Supplemental Tables 7 and 8). Sensitivity analyses conducted without an outlier did not change the direction or strength of the associations.

### Focus Group Discussions

**FIGURE 2:**
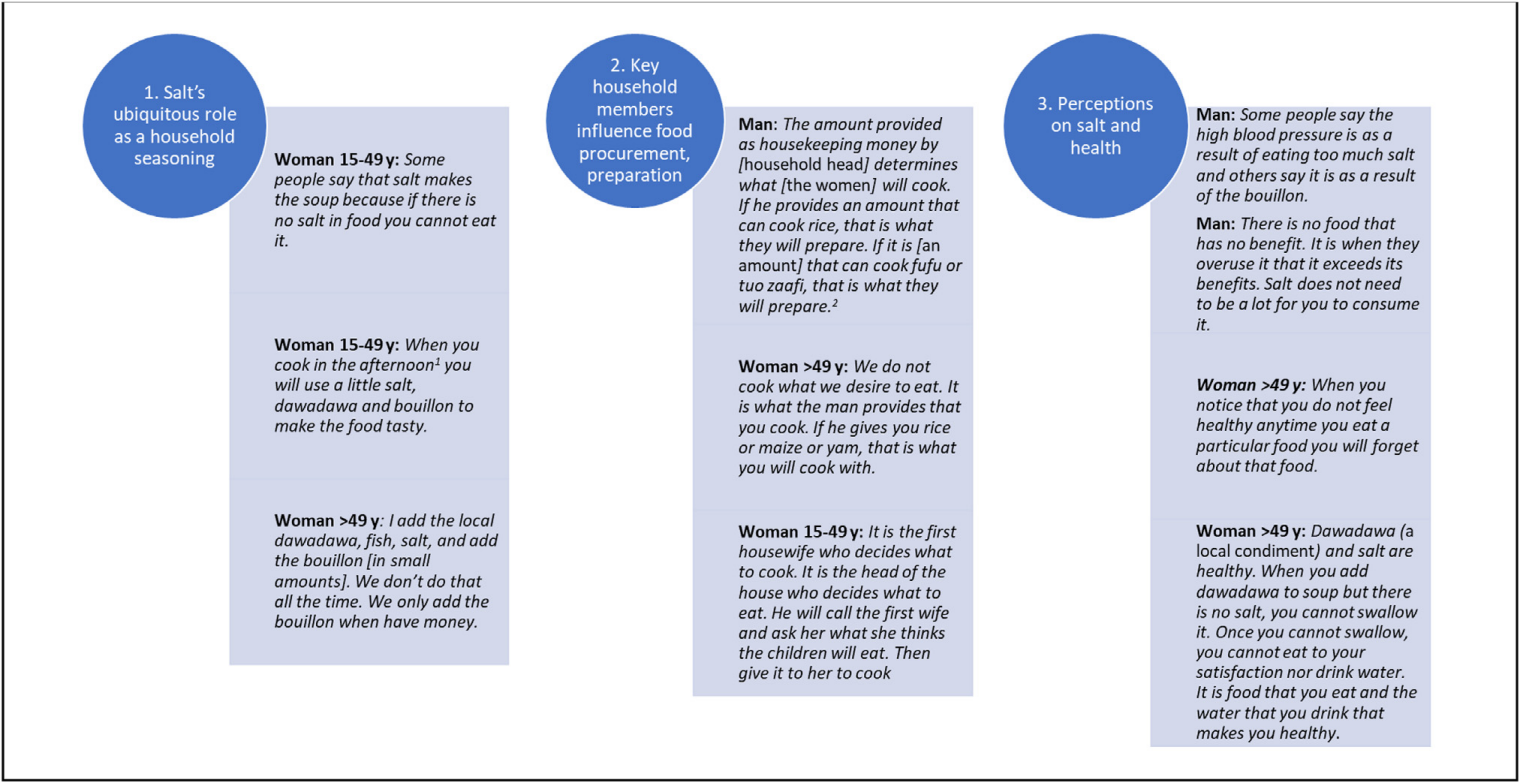
Exemplar quotes from participants of Focus Group Discussions in the Tolon and Kumbungu districts, northern Ghana: CoMIT project. Focus Group Discussions (*n* = 20) were conducted separately among women aged 15–49 y (*n* = 56), women >49 y (*n* = 29), and men aged ≥18 y (*n* = 29). Participant characteristics are listed in Supplemental Table 9. CoMIT, Condiment Micronutrient Innovation Trial. ^1^Cooking “in the afternoon” refers to cooking the evening meal if the household cooks once per day, or refers to lunch if the household cooks twice per day. ^2^Fufu is a dough most often made from pounded West African yam in the study area; tuo zaafi is boiled dough made from cereal flour (mainly maize and cassava in this region). These items are served with soups.

We further explored factors influencing household salt consumption through FGDs with women aged 15–49 y (*n* = 56 total participants), men aged ≥18 y (*n* = 29), and women aged >49 y (*n* = 29). Participant characteristics are presented in Supplemental Table 9. The salient influencing factors fell into 3 overarching themes: 1) salt’s ubiquitous role as a primary seasoning; 2) the influence of key household members on food procurement and preparation; and 3) perceptions of salt and health. Themes and supporting quotes are summarized in Figure 2.

#### Theme 1: Salt’s ubiquitous role as a primary seasoning.

When participants spoke of salt, they spoke of it in terms of its ubiquity in all cooking. Although other seasonings were also commonly added to various household cooked dishes, such as fish flakes, *dawadawa*, bouillon, or hot peppers, salt was used consistently in all cooking, including when other seasonings were unavailable, unaffordable, or not preferred by a household member. Participants also reported that food would be inedible if prepared without salt.

#### Theme 2: Influence of key household members on food procurement and preparation.

We asked participants how decisions were made about which dishes to prepare each day, and their responses exemplified how key household members influenced household food procurement and preparation. Participants reported that whoever provided the money for food procurement (often the household head) had a say in which foods were prepared. Participants from all FGDs further relayed that the taste preferences of the household head often dictated which foods were prepared by household cooks, with some participants stating the preferences of children were also considered. The taste preferences of household women appeared to have less influence than those of other household members.

#### Theme 3: Perceptions of salt and health.

Participants’ perceptions of salt and health were generally related to salt’s relationship with blood pressure and salt’s effect on food palatability. Eighty-one participants (71%), and ≥1 participant in each of the 20 FGDs (data not shown), reported that high blood pressure and stroke were common afflictions within their communities, and they discussed how overconsumption of salt and bouillon may contribute to these conditions. Participants appeared to determine the healthfulness of salt or bouillon based on how their bodies felt after its consumption. For example, if a harmful effect is observed after consuming salt, a person should reduce or stop its consumption. Salt was also often referred to as healthy due to its natural origins (i.e., not made with unknown ingredients), being part of their dietary tradition, and because it helped food become palatable, which was thought to increase the likelihood a person would eat and be better nourished. Finally, some participants reported confusion with differing messages of how salt consumption may relate to health outcomes, such as public health messaging to consume iodized salt alongside messaging to reduce overall salt intake.

## Discussion

In this mixed-methods study from 2 districts in Northern Region, Ghana, we analyzed quantitative data from a pilot survey that included adult women and young children and qualitative data from FGDs that included perspectives from both women and men. From reported household purchase data, we found that estimated consumption of salt, calculated by combining discretionary salt (table salt) and salt from bouillon, appeared to exceed global recommendations for salt intake for many children and most women. Bouillon contributed <25% to households’ daily total salt consumption; one-third of children and 50% of women appeared to exceed global recommendations from discretionary salt alone. Mean urinary sodium excretion and mean sodium-to-potassium ratio among WRA also suggested high sodium exposure. We identified a limited number of factors associated with household salt consumption, consistent with qualitative data emphasizing the ubiquity of salt as a household seasoning. Other salient qualitative themes illustrated how household salt consumption was influenced by the taste preferences of key household members, those providing money for food purchases, and perceptions of salt and health.

Relatively few household, individual, or KAP factors were associated with household salt consumption. However, some comments in the FGDs suggested the taste preferences of men and children in the household were of greater influence on food preparation (and thus household salt consumption) than those of women. It is possible that household characteristics such as poverty (and associated constraints on food purchases) or low education level of household members may contribute to salt intake; however, the data did not support this observation, possibly because of limited variation in the sample population. Surprisingly, both greater food insecurity and greater household assets were associated with greater household salt consumption. One might expect that greater food insecurity would reflect fewer resources available for food purchases, such as salt. However, FGD comments highlighted salt’s necessity as a seasoning and that when finances were constrained, purchasing salt was prioritized over buying other more expensive seasonings, such as bouillon. This finding suggests that household food insecurity may have a greater influence on salt consumption behaviors in this population than household assets (which aligns with the nonsignificant associations between household assets and total salt consumption and household assets and food insecurity). It suggests that interventions aimed at salt reduction do not need to prioritize specific population subgroups within this area. This finding might also reflect measurement error in estimating household salt consumption (including differential measurement error by purchasing amount and/or frequency) or reporting bias, but we found no significant correlations between household total salt consumption and social desirability variables. We did find that social desirability correlated with some KAP characteristics, which may indicate an awareness to respond “correctly” about reducing dietary salt. However, our estimates of high salt consumption in this population suggest that awareness is not sufficient to achieve (or report) salt consumption below global recommendations.

Strengths of our study include the use of mixed-methods methodology that explained convergent (such as overall high salt consumption) and divergent (such as greater salt consumption with greater food insecurity) quantitative and qualitative findings [54]. The face validity and reliability of the qualitative data were enhanced through triangulation with notes from the FGDs and discussions with fieldworkers, independent FGD transcript transcription and translation, independent transcript double-coding verified with Cohen’s Kappa, and the inclusion of exemplar quotes to illustrate and support each salient FGD theme [28,54]. Additionally, our estimates of salt consumption align with other salt consumption estimates in Ghana, with research specifying discretionary salt as the main dietary source of sodium and that salt is mainly added during cooking [14,20,60].

Limitations of our study include the use of household purchase data to generate salt consumption estimates [61], which did not include the contribution of salt from processed foods or foods eaten outside the home, and thus, we likely underestimated salt consumption. Incorporating these foods would tend to increase estimated salt consumption, which supports our conclusion that salt consumption exceeds global recommendations. The AME method used to estimate individual consumption from household data does not account for salt that is purchased but not necessarily consumed (e.g., used for food processing). The AME methodology assumes that intrahousehold food sharing is proportional to individual energy requirements [45], ignoring sex and age inequities where women and children may not consume their “fair share” of household meals [62]. However, because salt is used primarily in cooking family meals, it may be less subject to intrahousehold allocation errors than specific food items, such as snacks purchased outside the home. Although household purchase data are useful for assessing food consumption patterns, the data cannot provide accurate assessments of individual food intake, such as dietary data from 24-h recalls. But, even in the format of a structured 24-h recall, salt intake can still be misestimated as it is often added as a “dash” or out of habit and may not be accurately recalled. Thus, although the results do not represent precise estimates of individual salt intake (and are likely underestimates), they are nevertheless useful for a general assessment of salt intake in relation to the threshold of 5 g/d [63,64]. Another limitation was the use of a prediction equation to estimate salt intake from urinary sodium excretion, as prediction equations introduce bias and inconsistently over- or underpredict salt intake [48,51]. Using multiple 24-h urine collections (the gold standard) would better estimate total salt intake, but we believe this method would not change our conclusions regarding overall high salt exposure in this population. Additionally, the WHO recommends using spot urine samples to predict salt intake above or below the 5-g threshold at the population level [50]; therefore, we limited our interpretation of these data to the population level.

Given that high salt consumption coexists with micronutrient deficiencies in this region [26] and globally [65], salt reduction strategies must consider the fortification status of salt and salt-containing condiments like bouillon. In Ghana, most households use iodized salt [10]; thus, if consumption of a fortification vehicle (e.g., salt or bouillon) is reduced, there are implications for the impact of a fortification program on micronutrient status. However, others have argued that salt reduction and fortification strategies can concurrently pursue both aims [66]. For example, fortification programs can be designed (i.e., setting fortification levels of the fortification vehicle) and adjusted based on program monitoring and dietary intake data, and at the industry level, fortified salty condiments can be reformulated with lower sodium content, which has shown promising results in South Africa [67]. As salt reduction policies advance, developing and leveraging relationships between the public health, industry, and research sectors will be critical for success [68].

In this study population, the greatest challenge to salt reduction may be addressing salt’s ubiquity as a household seasoning and the taste preferences of influential household members. The majority of study participants reported awareness of the relationship between salt overconsumption and risk of developing hypertension or stroke. Thus, multipronged salt reduction strategies that combine public health messaging (e.g., social and behavioral change communication campaigns) that encourage consumption of iodized salt (or other fortified salty condiments like bouillon) and overall sodium reduction alongside large-scale interventions such as food fortification and product reformulation are likely necessary to address both high salt consumption and micronutrient deficiency in this population.

In this mixed-methods study from northern Ghana, we found that estimated daily consumption of salt, including discretionary salt and salt from bouillon, appeared to exceed global recommendations for many young children and most adult women, a likely underestimation of salt intake as the contribution of processed foods or foods consumed outside the home were not included. The vast majority of salt was consumed as discretionary salt, with bouillon contributing <25%, reducing the relevance of one argument against considering bouillon as a micronutrient fortification vehicle. Multipronged, broadly targeted salt reduction strategies that include messaging linking salt consumption to health outcomes may be useful salt reduction approaches in this region.

## Acknowledgments

We gratefully acknowledge the contributions of UCSF MLK Cores Research Facility (https://mlkcores.ucsf.edu) for support with elemental analysis; Ahmed Fuseini and Ebenezer Adjetey for their contributions to study implementation; and Dr. Francene Steinberg, Dr. Christine Stewart, and Dr. Anne Williams for their critical manuscript review and commentaries. We also sincerely thank the fieldworkers, survey personnel, and participants who made this research possible.

## Author contributions

The authors’ responsibilities were as follows – JND, RES, SAA: designed the research; SMK, KWN conducted the study; JND, SMK, CDA, XT, KWN, RES: analyzed data; SMK, CD, KRW, EB, KPA, SV, RES: provided critical feedback; JND: wrote the paper; RES: had primary responsibility for final content; and all authors: read and approved the final manuscript.

### Conflict of interest

JND: WHO (consulting); KA: WHO (advisory relationship and travel reimbursement); RE-S: Financial support from Helen Keller International and the University of California, Davis, Gifford Center for Population Studies; advisory relationship and travel reimbursement from the WHO. RE-S is an Academic Editor for Current Developments in Nutrition and played no role in the Journal’s evaluation of the manuscript. All other authors report no conflicts of interest.

## Funding

This work was supported, in whole, or in part, by the Bill & Melinda Gates Foundation [INV-007916]. Under the grant conditions of the Foundation, a Creative Commons Attribution 4.0 Generic License has already been assigned to the Author Accepted Manuscript version that might arise from this submission.

## Data availability

Consistent with policy of the Bill & Melinda Gates Foundation, data and forms will be made available online at osf.io/t3zrn/ 3 y after the completion of data collection.
