# Supplementary material for: Consumption of Discretionary Salt and Salt from Bouillon among Households, Women, and Young Children in Northern Region, Ghana: A Mixed-Methods Study with the Condiment Micronutrient Innovation Trial (CoMIT) Project
Source: Curr Dev Nutr. 2024 Feb 6;8(3):102088. doi: 10.1016/j.cdnut.2024.102088 (PMC10897853; doi:10.1016/j.cdnut.2024.102088)
Supplement: Multimedia component 1 [file mmc1.docx]

Supplemental Material Table of Contents

[SUPPLEMENTAL METHODS 3](#_Toc155687245)

[Overview 3](#_Toc155687246)

[Participants, eligibility, and consent 3](#_Toc155687247)

[COVID-19 screening protocol 3](#_Toc155687248)

[Sampling and recruitment 3](#_Toc155687249)

[Additional quantitative data collection details 4](#_Toc155687250)

[Anthropometric measurements 4](#_Toc155687251)

[BMI definitions 4](#_Toc155687252)

[Blood pressure measurements and definitions 4](#_Toc155687253)

[**Supplemental Table 1.** Blood pressure and hypertension guidelines for women of reproductive age (15-49 y) 5](#_Toc155687254)

[**Supplemental Table 2.** ‘At risk’ blood pressure guidelines for preschool-aged children (2-5 y) 5](#_Toc155687255)

[Urine sample collection and laboratory analysis 5](#_Toc155687256)

[Adult Male Equivalent (AME) method 6](#_Toc155687257)

[Estimating WRA daily total salt consumption from spot urine samples 6](#_Toc155687258)

[Exploratory analysis with KAP-related factors 6](#_Toc155687259)

[ADDITONAL SUPPLEMENTAL TABLES 7](#_Toc155687260)

[**Supplemental Table 3.** List of potential predictors of household salt consumption and social desirability variables 7](#_Toc155687261)

[**Supplemental Table 4.** Intercoder reliability score (ICR) calculated as Cohen’s Kappa and percent agreement 9](#_Toc155687262)

[**Supplemental Table 5.** Factors associated with household daily discretionary salt consumption among households who participated in the pilot survey 10](#_Toc155687263)

[**Supplemental Table 6.** Knowledge, attitude, and practice factors associated with household daily discretionary salt and total salt consumption among households who participated in the pilot survey 14](#_Toc155687264)

[**Supplemental Table 7.** Estimated per capita discretionary salt consumption (g/d) by asset quintiles and food insecurity status among households participating in the pilot survey 16](#_Toc155687265)

[**Supplemental Table 8.** Salt quantity (g) last purchased by asset quintiles among households participating in the pilot survey 16](#_Toc155687266)

[**Supplemental Table 9.** Characteristics of focus group participants 16](#_Toc155687267)

[SUPPLEMENTAL FIGURES 17](#_Toc155687268)

[**Supplemental Figure 1.** Distributions of household discretionary salt and total salt consumption (g/d) before and after replacement of values below 2.5 percentile and above 97.5 percentile among participating households in the pilot survey 17](#_Toc155687269)

[**Supplemental Figure 2.** Correlations between potential predictors of household salt consumption, including social desirability (SD) variables, among participants in the pilot survey 18](#_Toc155687270)

[**Supplemental Figure 3.** Flow chart of participating households, participants, and data collected in the pilot survey and focus group discussions 19](#_Toc155687271)

[**Supplemental Figure 4.** Factors associated with household salt consumption among women in the pilot survey from mixed effect ANOVA analyses 20](#_Toc155687272)

[SUPPLEMENTAL APPENDIX 21](#_Toc155687273)

[Focus Group Discussion (FGD) Guide 21](#_Toc155687274)

[SUPPLEMENTAL REFERENCES 24](#_Toc155687275)

# SUPPLEMENTAL METHODS

## Overview

The main objective of the pilot survey was to evaluate the micronutrient status and assess intake and usage patterns of fortified foods among 3 priority populations: non-pregnant non-lactating women of reproductive age (WRA, 15-49 y), non-pregnant lactating women of reproductive age 15-49 y and 4-18 months post-partum (LW), and children 2-5 y. The pilot survey and focus group discussions (FGDs) took place November 2020 to March 2021. The results of the pilot survey and FGDs were used to inform a planned randomized controlled trial (RCT) in the same area.

## Participants, eligibility, and consent

The 3 priority groups were chosen due to their greater physiological demands for micronutrients and greater risk of micronutrient deficiency (1), and because these same physiologic groups were planned for the RCT. For the pilot survey, potential participants were excluded if they suffered from a chronic severe medical condition, were ill (i.e., fever, diarrhea) ≤24-h prior to any research activities, or did not pass COVID-19 screening (see next section). Informed written (or thumbprint) consent was obtained from eligible participants, or their parent/guardian or caregiver (children and women 15-17 years of age and not married, divorced, separated, or living with a partner), with community members not affiliated with the study acting as witnesses for participants unable to read or write. FGD participants (WRA, men ≥18 y, and women >49 y) were eligible if they provided written informed consent and screened negative for COVID-19. All participants received a bar of Key Soap as an incentive for each day of survey activities.

## COVID-19 screening protocol

At the time of any initial interaction with participants (i.e., at recruitment or data collection if they occurred on different days), participants were screened for COVID-19 following the latest protocols from Ghana Health Service (GHS) and the World Health Organization (WHO). The study COVID-19 screening protocol included checking each participant’s temperature twice, and asking if they were aware of any close contact with a COVID-19 positive person (CPP) in the previous 2 weeks. Close contact was defined as: 1) having lived or stayed overnight with the CPP; 2) had intimate relations with the CPP; 3) took care of the CPP or the CPP took care of the participant; 4) stayed within 2 meters of the CPP for more than 10 minutes with the CPP not wearing a mask; 5) were exposed to direct contact with body fluids of the CPP) (2). During all research activities, fieldworks, participants, and any accompanying family members were required to sanitize their hands and wear mask (adults only) that completely covered their nose and mouth. Masks were provided to anyone in need.

## Sampling and recruitment

Within the 2 districts, clusters (e.g., villages, towns, communities) were selected through consultation with district maps. Within the selected clusters, fieldworkers identified households with potential participants through a random walk method with door-to-door recruitment starting from a random central location. Selected households in which 1 or more members of the 3 physiological groups resided were eligible for recruitment, or one eligible individual was randomly chosen based on a Kish Table if a household contained more than 1 eligible participant per physiologic group. Recruitment of FGD participants followed the same sampling protocol except that only 1 group (WRA, men, or women >49 y) was recruited per household.

For the pilot survey, sample size was determined based on the main outcome of micronutrient status, with an assumed estimated prevalence of micronutrient deficiency of 50% for any one micronutrient included in the proposed multiple micronutrient fortified bouillon cubes (iron, folic acid, zinc, vitamin A, and vitamin B12). Per this calculation, 250 participants per physiological group allowed for analysis precision up to ±7%, including 20% potential loss to attrition.

## Additional quantitative data collection details

### Anthropometric measurements

Among WRA and children only, anthropometric measurements of standing height and weight (with participants lightly clothed) were completed by trained fieldworkers on equipment calibrated daily (Seca® 217 stadiometer and Seca® 874 scale measured to 0.1 cm and 0.5 kg precision, respectively). Measurements were completed in triplicate and results averaged.

### BMI definitions

Among WRA, Body Mass Index (BMI) categories were defined as underweight (BMI <18.5 kg/m^2^), normal weight (BMI ≥18.5-24.9 kg/m^2^), overweight (BMI ≥25.0-29.9 kg/m^2^), or obesity (BMI ≥30.0 kg/m^2^) (5). Among children, BMI for age z-score (BAZ) categories were defined as underweight (BAZ <-2 SD), normal weight (BAZ -2 ≥ SD ≤ 2), or at risk of overweight (BAZ >2 SD). Also among children, stunting was defined as height-for-age z-score <-2 SD, and wasting as weight-for-height z-score <-2 SD. Anthropometric z-scores were calculated according to WHO standard (3).

### Blood pressure measurements and definitions

Blood pressure was measured among women and children in triplicate with measures one minute apart (results averaged) using a Riester® RBP-100 automatic portable upper-arm blood pressure monitor (Jungingen, Germany). Participants sat quietly for five minutes prior to the first measurement, and the circumference of participants’ upper arms was measured to select the appropriate cuff size (4).

Among WRA, elevated blood pressure and hypertension were defined according to three organizations to capture a broader picture of hypertension risk: the American Heart Association (5), the International Society of Hypertension (6), and the WHO (7). For children, ‘at risk’ blood pressure thresholds were defined by American Academy of Pediatrics (8).

### **Supplemental Table 1.** Blood pressure and hypertension guidelines for women of reproductive age (15-49 y)

| **Organization** | **Normal blood pressure (mmHg)** | **Elevated blood pressure (mmHg)** | **Hypertension (mmHg)** |
| --- | --- | --- | --- |
| American Heart Association | SBP <120 and DBP <80 | SBP 120-129 and DBP <80 | SBP ≥130 or DBP ≥80 |
| International Society of Hypertension | SBP <130 and DBP <85 | SBP 130-139 and/or DBP 85-89 | SPB ≥140 and/or DBP ≥90 |
| World Health Organization | SBP <120 and DBP <80 |  | SPB ≥140 and/or DBP ≥90 |

**Supplemental Table 1 Footnotes:** International Society of Hypertension classifies ‘Elevated blood pressure’ as ‘High-normal blood pressure’. References: American Heart Association (5); International Society of Hypertension (6); WHO (7). DBP, diastolic blood pressure; SBP, systolic blood pressure.

### **Supplemental Table 2.** ‘At risk’ blood pressure guidelines for preschool-aged children (2-5 y)

| **Age, y** | **Males** | **Females** |
| --- | --- | --- |
| 2 | SBP 100 or DBP 55-57 | SBP 101 or DBP 58-59 |
| 3 | SBP 101 or DBP 58-60 | SBP 102 or DBP 60-61 |
| 4 | SBP 102 or DBP 60-62 | SBP 103 or DBP 62-63 |
| 5 | SBP 103 or DBP 63-65 | SBP 104 or DBP 64-66 |

**Supplemental Table 2 Footnotes:** ‘At risk’ refers to the screening threshold at which children aged 2-5 y should undergo further evaluation for elevated blood pressure by a qualified professional, according to the American Academy of Pediatrics (8). DPB, diastolic blood pressure; SBP, systolic blood pressure.

### Urine sample collection and laboratory analysis

From each WRA, one spot urine sample was collected to measure urinary sodium, potassium, and creatinine. Urine samples (1.5 mL) were aliquoted and refrigerated in the field, and then frozen and stored at -20°C for transport to the University of California, San Francisco, Martin Luther King, Jr Research Facility for analysis.

Urinary sodium and potassium concentrations were determined by inductively-coupled plasma spectrometry (ICP-OES). Urine samples were thawed, vortexed, and 0.5ml was transferred into trace metal-free tubes. Urine samples were then dissolved into 0.25ml OmniTrace 70% HNO3 and digested overnight at 60°C. The acid lysates were then diluted to 5% HNO3 with OmniTrace water before analysis with an Agilent 5100 SVDV ICP-OES calibrated with National Institute of Standards and Technology (NIST)–traceable elemental standards and routinely validated with Seronorm Trace Element Serum Levels 1 and 2. A random selection of samples (n=24, 10%) were run as technical replicates at the beginning, middle, and end of the analysis period (coefficient of variation for sodium: 7.9%; potassium: 3.2%). Urine creatinine was measured with the Cayman creatinine colorimetric assay kit (#500701). The urine samples were diluted 20-fold and analyzed in duplicate (coefficient of variation: -4.8%). High and low controls were included on each plate, and values were within reported ranges. The relative difference for all analytes was <1%.

### Adult Male Equivalent (AME) method

The AME method assigns an adjustment factor (AME unit) to each member of a household based on sex- and age-specific energy expenditure and energy requirements, where males 19-30 y have an AME value of 1.0 and are the reference category (i.e., AME=1 for adult males aged 19-30 y, AME=0.7 for adult females aged 19-30 y, and AME=0.5 for children). To estimate individual daily consumption of discretionary salt, we 1) calculated a household AME value by summing all AME units from all household members; 2) calculated the individual AME fraction attributed to WRA and children by dividing each individual AME value by the household AME value; and 3) calculated the individual daily salt consumption by multiplying the individual AME fraction by the household discretionary salt consumption (g/d) (9). This process was repeated for estimating individual consumption of total salt with an additional step of multiplying the final answer by 55%. Final estimates for WRA and children are reported as g/d/AME.

### Estimating WRA daily total salt consumption from spot urine samples

From spot urine samples (WRA only), averages of replicate urinary sodium, creatinine, and potassium values were calculated with 1 value per participant included for analysis. The distributions of each analyte were examined and all observations retained. To estimate daily total salt consumption from spot urine samples, we first used the INTERSALT equation to predict 24-h urinary sodium excretion (10):

23 {5.07 + [0.34 * spot Sodium (mmol/L)] – [2.16 * spot Creatinine (mmol/L)] – [0.09 * spot Potassium (mmol/L)] + [2.39 * BMI (kg/m^2^)] + [2.35 x age (years)] – [0.03 * age^2^ (years)]}

To estimate daily total salt consumption (g/d), we then divided the predicted 24-h urinary sodium excretion (mg/d) by 390 as there are 390 mg sodium in 1 g sodium chloride (‘table salt’) (11).

### Exploratory analysis with KAP-related factors

In exploratory analyses, additional linear mixed-effects models were constructed to test associations between groups of KAP-related factors and estimated household discretionary and total salt consumption. In minimally-adjusted models, selected KAP factors were placed into 3 separate groups (i.e., separate groups of knowledge, attitude, and practice factors); factor placement into KAP groups was determined based on the intent of the question (12) **(Supplemental Table 3**). Each KAP group was tested separately with the two outcome variables (estimated household discretionary salt consumption and estimated household total salt consumption, continuous (g/d)). Then, marginally statistically significant factors (p<0.10) within each KAP group were included together in a multivariable mixed-effects KAP model; collinearity was again assessed with variance inflation factors (>5) and tolerance (<0.1).

# ADDITONAL SUPPLEMENTAL TABLES

## **Supplemental Table 3.** List of potential predictors of household salt consumption and social desirability variables

| **Predictor short name** | **Full description of predictor** | **Categories for regression analysis** |
| --- | --- | --- |
| *Household-level predictors* |  |  |
| Size | Household size (number of members) | 1-8 members  9-11 members  12-15 members  16+ members |
| District | District of Tolon or Kumbungu | Tolon  Kumbungu |
| Setting | Urban or rural setting | Urban  Rural |
| Participant type | Woman of reproductive age (15-49 y, WRA) or lactating woman (LW) | WRA  Lactating woman |
| Asset quintiles | Asset quintiles (1^st^ lowest, 5^th^ highest) | 1st (low), 2, 3, 4, 5^th^ (high) |
| Food insecurity | Food insecurity status: None, mild, moderate, severe | None to mild  Moderate  Severe |
| Education level | Highest level of education completed by the household head (none, primary school, secondary+) | None  Preschool or primary  Secondary or greater |
| Cons. of DD | Household consumption of *dawadawa* where the cutoffs for each category were defined by the natural cutoffs present in the variable’s distribution. | Low (0-10 g/d)  Medium (10.01-48.9 g/d)  High (>49 g/d) |
| *Individual-level predictors* | *[individual-level: WRA and LW]* |  |
| Age | Age in years of WRA or LW | 15-24 y  25-34 y  35-49 y |
| Hx of heart dis. | History of heart disease (self report) | No, Yes |
| Hypertension | Current hypertension (self report) | No, Yes |
| BMI | Standard BMI categories | Normal (18.5-24.9 kg/m^2^)  Underweight (<18.5 kg/m^2^)  Overweight/obese (≥25.0 kg/m^2^) |
| *Dietary patterns (individual-level)* | *[individual-level: WRA and LW]* |  |
| Vegetable cons. | Frequency of consuming vegetables, days per week | 1-2 d/wk  3-5 d/wk  6-7 d/wk |
| Fruit cons. | Frequency of consuming fruit, days per week | None  1-2 d/wk  ≥3 d/wk |
| Salty snacks | Frequency of consuming salty snacks such as crisps, days per week | None  1-2 d/wk  ≥3 d/wk |
| Bouillon cons. | Frequency of consuming foods prepared with bouillon, days per week | 0-6 d/wk  7 d/wk |
| *Knowledge predictors* | *[individual-level: WRA and LW]* |  |
| Qty salt | How much salt do you think you consume? | Just the right amount  Too much  Too little |
| Salt as a problem | Do you think that too much salt in your diet could cause a health problem? | No  Yes  Don’t know |
| *Attitude predictors* | *[individual-level: WRA and LW]* |  |
| Lower salt | How important to you is lowering salt in your diet? | Somewhat important  Very Important  Not at all important |
| Benefits of salt | Do you think that having salt in your diet is good, bad, or has no effect? | No effect  Good  Bad |
| Benefits of DD | Do you think that having dawadawa in your diet is good, bad, or has no effect? | No effect  Good  Bad |
| Benefits of bouillon | Do you think having bouillon in your diet is good, bad, or has no effect? | No effect  Good  Bad |
| *Practice predictors* | *[individual-level: WRA and LW]* |  |
| Add salt at table | Do you add salt to your food at the table always, sometimes, or never? | Always/often/sometimes  Rarely/never |
| Add salt in cooking | Do you add salt to your food while cooking always, sometimes, or never? | Always/often/sometimes  Rarely/never |
| Eat proc. foods | Do you eat salty processed foods always, sometimes or never? | Always/often/sometimes  Rarely/never |
| Cntl salt intake | Do you do at least 1 action to control your salt intake? | No, Yes |
| *Social desirability* | *[individual-level: WRA and LW]* | *(not included in regression analysis)* |
| SD1 | Social desirability 1: Do you occasionally give up doing something because you don’t think you have the ability? | No, Yes |
| SD2 | Social desirability 2: Do you occasionally feel like not listening to people even if you know they are right? | No, Yes |
| SD3 | Social desirability 3: Are you sometimes irritated/annoyed by people who ask you to do something for them? | No, Yes |
| SD4 | Social desirability 4: Are you always courteous, even to people who are disagreeable/not pleasant? | No, Yes |
| SD5 | Social desirability 5: When you make a mistake, are you always willing to admit it? | No, Yes |

**Supplemental Table 3 Footnotes**: All analyses were completed with categorical predictors. See main text and supplemental methods for references and further descriptions of predictors. Social desirability questions taken from the Marlowe-Crowne social desirability scale (13) and were not predictors in the linear regression models but were used in correlation sensitivity analyses. All predictors were selected *a priori* according to our statistical analysis plan.

## **Supplemental Table 4.** Intercoder reliability score (ICR) calculated as Cohen’s Kappa and percent agreement

| **Segment** | **Average ICR** | **Percent Agreement** |
| --- | --- | --- |
| 1. Household makeup; household members; meal sharing | 0.94 | 95.9% |
| 2. Cooking decisions and cooking duties; sharing of cooking during | 0.97 | 97.8% |
| 3. Bouillon preferences and habits | 0.94 | 96.1% |
| 4. Bouillon usage and consumption | 0.92 | 95.0% |
| 5. Other flavorings and seasonings, including salt and *dawadawa* | 0.93 | 96.4% |
| 6. Bouillon purchasing | 0.95 | 96.8% |
| 7. Health and nutritional problems | 1.00 | 100% |
| 8. Description of fortified bouillon cubes | 0.88 | 91.4% |
| 9. Knowledge and usage of fortified bouillon cubes | 1.00 | 100% |
| 10. Beliefs about fortified bouillon cubes | 0.91 | 95.1% |
| 11. Perceptions of salt, *dawadawa*, and MSG | 1.00 | 100% |
| **Average total** | **0.95** | **96.5%** |

**Supplemental Table 4 Footnotes:** ICR score calculated in Stata 16. Average ICR represents the average final ICR of n=7 focus group discussion (FGD) transcripts that were independently coded by 2 coders. See FGD guide in Supplemental Appendix for full descriptions of FGD questions (segments).

**Supplemental Table 5.** Factors associated with household daily discretionary salt consumption among households who participated in the pilot survey^1^

|  | **Variable** | **Category** | **HH discretionary salt consumption, g/d** | | **Minimally adjusted analyses^2^** | | **Multivariable analyses^2^** | |
| --- | --- | --- | --- | --- | --- | --- | --- | --- |
|  |  |  | n | Mean (SD) | *β* (95% CI) | *p* | *β* (95% CI) | *p* |
| *Household-level factors* | | | |  |  |  |  |  |
|  | Size, # of members | 1-8 | 118 | 53.1 (52.7) | ref. | ***<0.001*** | ref. | ***0.001*** |
|  |  | 9-11 | 135 | 60.9 (45.4) | 6.9 (-6.8, 20.4) |  | -1.1 (-14.9, 12.7) |  |
|  |  | 12-15 | 112 | 74.8 (55.7) | 25.6 (10.9, 40.3) |  | 7.7 (-8.7, 24.2) |  |
|  |  | 16+ | 112 | 104.4 (70.1) | 49.9 (35.4, 64.5) |  | 32.3 (14.9, 49.6) |  |
|  |  |  |  |  |  |  |  |  |
|  | District | Tolon | 242 | 76.1 (64.8) | ref. | *0.100* | ref. | *0.600* |
|  |  | Kumbungu | 235 | 68.6 (52.7) | -10.0 (-21.9, 1.9) |  | 3.5 (-9.7, 16.8) |  |
|  |  |  |  |  |  |  |  |  |
|  | Setting | Urban | 232 | 64.7 (53.4) | ref. | ***0.006*** | ref. | *0.100* |
|  |  | Rural | 245 | 79.7 (63.5) | 16.6 (4.7, 28.5) |  | 11.0 (-2.1, 24.2) |  |
|  |  |  |  |  |  |  |  |  |
|  | Participant type | WRA | 239 | 71.4 (57.8) | ref. | *0.760* | ref. | *0.220* |
|  |  | Lactating women | 238 | 73.5 (60.7) | 1.5 (-8.3, 11.3) |  | 6.7 (-4.0, 17.5) |  |
|  |  |  |  |  |  |  |  |  |
|  | Asset quintiles | 1^st^ (lowest) | 88 | 52.6 (44.2) | ref. | ***0.060*** | ref. | *0.520* |
|  |  | 2^nd^ | 81 | 73.9 (61.2) | 15.4 (-1.4, 32.3) |  | 13.4 (-4.0, 30.7) |  |
|  |  | 3^rd^ | 95 | 86.0 (66.3) | 23.2 (6.5, 39.8) |  | 14.1 (-3.2, 31.4) |  |
|  |  | 4^th^ | 107 | 74.1 (58.3) | 7.2 (-9.8, 24.3) |  | 8.1 (-10.2, 26.4) |  |
|  |  | 5^th^ (highest) | 106 | 73.8 (59.7) | 13.5 (-4.6, 31.6) |  | 9.4 (-9.7, 28.6) |  |
|  |  |  |  |  |  |  |  |  |
|  | Food insecurity | None to mild | 113 | 63.7 (46.4) | ref. | ***<0.001*** | ref. | *0.050* |
|  |  | Moderate | 250 | 62.7 (51.4) | 3.1 (-8.9, 15.0) |  | -2.6 (-16.8, 11.5) |  |
|  |  | Severe | 114 | 102.5 (79.9) | 37.3 (23.5, 51.1) |  | 17.3 (-1.4, 36.1) |  |
|  |  |  |  |  |  |  |  |  |
|  | Hh head education | None | 110 | 69.9 (61.9) | ref. | *0.360* |  |  |
|  |  | Preschool or primary | 73 | 81.4 (64.5) | 11.2 (-5.4, 27.8) |  |  |  |
|  |  | Secondary or greater | 294 | 71.2 (56.8) | 2.1 (-11.1, 15.3) |  |  |  |
|  |  |  |  |  |  |  |  |  |
|  | Dawadawa consumption | Low | 104 | 63.6 (43.4) | ref. | *0.720* |  |  |
|  |  | Medium | 286 | 76.5 (63.3) | 4.5 (-8.3, 17.3) |  |  |  |
|  |  | High | 58 | 61.9 (62.6) | -0.4 (-18.5, 17.6) |  |  |  |
| *Demographic factors (individual-level)* | | | |  |  |  |  |  |
|  | Women age, y | 15-24 y | 129 | 71.7 (61.9) | ref. | *0.960* |  |  |
|  |  | 25-34 y | 206 | 75.0 (61.8) | -1.5 (-13.9, 10.9) |  |  |  |
|  |  | 35-49 y | 138 | 70.4 (53.1) | 0.1 (-13.2, 13.4) |  |  |  |
|  |  |  |  |  |  |  |  |  |
|  | History of heart disease | No | 438 | 71.5 (59.0) | ref. | *0.650* |  |  |
|  |  | Yes | 37 | 86.2 (61.3) | 4.3 (-14.3, 23.0) |  |  |  |
|  |  |  |  |  |  |  |  |  |
|  | Current hypertension | No | 203 | 73.1 (60.5) | ref. | *0.640* |  |  |
|  |  | Yes | 12 | 70.6 (38.5) | 7.8 (-24.7, 40.2) |  |  |  |
|  |  |  |  |  |  |  |  |  |
|  | BMI | Normal | 154 | 77.0 (59.7) | ref. | *0.990* |  |  |
|  |  | Underweight | 20 | 71.3 (59.1) | -0.7 (-26.2, 24.8) |  |  |  |
|  |  | Overweight/obese | 43 | 58.8 (57.2) | -14.2 (-32.7, 4.2) |  |  |  |
| *Dietary patterns (individual-level)* | | | | |  |  |  |  |
|  | Vegetable consumption | 1-2 d/wk | 57 | 50.6 (49.9) | ref. | ***<0.001*** | ref. | ***0.030*** |
|  |  | 3-5 d/wk | 215 | 61.8 (51.3) | 10.2 (-5.4, 25.7) |  | 9.7 (-9.2, 28.5) |  |
|  |  | 6-7 d/wk | 203 | 90.2 (64.7) | 39.1 (22.4, 55.8) |  | 24.6 (-6.8, 34.5) |  |
|  |  |  |  |  |  |  |  |  |
|  | Fruit consumption | None | 126 | 54.8 (48.3) | ref. | ***<0.001*** | ref. | *0.350* |
|  |  | 1-2 d/wk | 285 | 74.2 (60.0) | 14.9 (3.4, 26.4) |  | 1.5 (-12.3, 15.4) |  |
|  |  | ≥3 d/wk | 64 | 100.8 (64.2) | 34.3 (17.3, 51.2) |  | 13.9 (-6.8, 34.5) |  |
|  |  |  |  |  |  |  |  |  |
|  | Salty snacks consumption | None | 165 | 66.1 (49.5) | ref. | *0.990* |  |  |
|  |  | 1-2 d/wk | 225 | 74.8 (66.5) | -1.1 (-15.2, 12.9) |  |  |  |
|  |  | ≥3 d/wk | 85 | 79.5 (55.0) | -0.9 (-17.2, 15.5) |  |  |  |
|  |  |  |  |  |  |  |  |  |
|  | Consumption of foods made with bouillon | 0-6 d/wk | 27 | 40.6 (24.3) | ref. | ***0.050*** | ref. | *0.350* |
|  |  | 7 d/wk | 311 | 71.3 (58.4) | 21.3 (0.2, 42.4) |  | 9.4 (-10.4, 29.2) |  |
| *Knowledge factors (individual-level)* | | | | |  |  |  |  |
|  | Quantity of salt consumed | Just the right amount | 368 | 72.7 (57.3) | ref. | *0.830* |  |  |
|  |  | Too much | 25 | 76.2 (50.7) | 1.3 (-21.1, 23.7) |  |  |  |
|  |  | Too little | 82 | 71.0 (70.1) | 4.2 (-9.2, 17.5) |  |  |  |
|  |  |  |  |  |  |  |  |  |
|  | Think dietary salt causes health problems | No | 43 | 51.0 (39.9) | ref. | ***0.048*** | ref. | *0.190* |
|  |  | Yes | 377 | 76.6 (60.9) | 22.0 (4.4, 39.6) |  | 18.6 (-2.7, 40.0) |  |
|  |  | Don’t know | 55 | 61.9 (56.1) | 16.9 (-5.1, 38.8) |  | 21.5 (-4.1, 47.1) |  |
| *Attitude factors (individual-level)* | | | | |  |  |  |  |
|  | Importance of lowering salt in the diet | Somewhat important | 170 | 69.1 (56.9) | ref. | ***<0.001*** | ref. | ***0.030*** |
|  |  | Very important | 190 | 56.7 (44.3) | -12.9 (-24.0, -1.9) |  | -3.07 (-16.8, 10.6) |  |
|  |  | Not at all important | 115 | 104.2 (71.4) | 26.45 (13.7, 39.3) |  | 20.9 (3.6, 38.2) |  |
|  |  |  |  |  |  |  |  |  |
|  | Perception of dietary salt | No effect | 91 | 63.7 (42.2) | ref. | *0.320* |  |  |
|  |  | Good | 144 | 77.5 (62.6) | 7.1 (-7.1, 21.3) |  |  |  |
|  |  | Bad | 103 | 61.3 (59.0) | -3.3 (-18.4, 11.3) |  |  |  |
|  |  |  |  |  |  |  |  |  |
|  | Perception of dietary dawadawa | No effect | 32 | 64.2 (49.4) | ref. | *0.840* |  |  |
|  |  | Good | 306 | 69.3 (57.8) | 2.1 (-18.3, 22.5) |  |  |  |
|  |  | Bad | 0 | -- | -- |  |  |  |
|  |  |  |  |  |  |  |  |  |
|  | Perception of dietary bouillon | No effect | 48 | 76.3 (57.0) | ref. | *0.230* |  |  |
|  |  | Good | 231 | 67.1 (58.4) | -14.1 (-30.8, 2.6) |  |  |  |
|  |  | Bad | 59 | 69.4 (51.8) | -7.6 (-28.0, 12.7) |  |  |  |
| *Practice factors (individual-level)* | | | | |  |  |  |  |
|  | Add salt at the table | Always/often/sometimes | 196 | 85.2 (61.9) | ref. | ***<0.001*** | ref. | *0.410* |
|  |  | Rarely/never | 279 | 63.8 (55.8) | -14.2 (-24.4, -4.0) |  | -5.0 (-17.0, 7.0) |  |
|  |  |  |  |  |  |  |  |  |
|  | Add salt during cooking | Always/often/sometimes | 454 | 72.4 (60.0) | ref. | *0.510* |  |  |
|  |  | Rarely/never | 21 | 77.6 (40.2) | 8.1 (-16.0, 32.3) |  |  |  |
|  |  |  |  |  |  |  |  |  |
|  | Eat processed foods | Always/often/sometimes | 233 | 69.6 (62.8) | ref. | *0.400* |  |  |
|  |  | Rarely/never | 242 | 75.5 (55.6) | 4.4 (-5.8, 14.5) |  |  |  |
|  |  |  |  |  |  |  |  |  |
|  | Do at least 1 action regularly to control dietary salt | No | 91 | 74.2 (48.6) | ref. | *0.120* |  |  |
|  |  | Yes | 384 | 72.2 (61.6) | -10.4 (-23.1, 2.2) |  |  |  |

^1^ Discretionary salt refers to ‘table salt’ and estimates of Hh salt consumption were calculated from purchase data using the Fortification Assessment Coverage Toolkit (14). Hh discretionary salt consumption (g/d) presented as unadjusted mean (SD). Total households included: n=363. Hh head education defined as the highest education level completed. Hh consumption of dawadawa categories defined as: low, 0-10 g/d; med, 10.01-48.9 g/d; high >49 g/d, where the cutoffs for each category were defined by the natural cutoffs present in the variable’s distribution. History of heart disease was self-reported. Hypertension defined according to the WHO definition (7). Salty snacks defined as salty crisps, chips, nuts, or salty fried foods eaten between main meals. BMI, body mass index; Hh, household; CoMIT, Condiment Micronutrient Innovation Trial; ref., reference group; WHO, World Health Organization.

^2^ Minimally adjusted and multivariable analyses were linear mixed effects regression models that controlled for Hh size, district (Tolon/Kumbungu), area (urban/rural), and participant type (woman of reproductive age or lactating woman) as fixed effects, and cluster as a random effect. The outcome variable (Hh discretionary salt consumption in g/d) was tested as a continuous variable and all predictors were categorical variables. Predictors were included from both the household-level and individual-level (from data from non-pregnant, non-lactating women of reproductive age, 15-49 y, and non-pregnant lactating women, 6-18 mos post-partum, 15-49 y; n=487 total). After being tested separately in minimally adjusted models, marginally significant predictors (p<0.1, **bolded**) were included in the multivariable model.

**Supplemental Table 6.** Knowledge, attitude, and practice factors associated with household daily discretionary salt and total salt consumption among households who participated in the pilot survey^1^

|  |  |  | Outcome:  Hh discretionary salt consumption, g/d | | Outcome:  Hh total salt consumption, g/d | |
| --- | --- | --- | --- | --- | --- | --- |
|  | Variable | Categories | KAP group minimally-adjusted model^2^ | KAP multivariable model^2^ | KAP group minimally-adjusted model^2^ | KAP multivariable model^2^ |
|  |  |  | *β* (95% CI) | *β* (95% CI) | *β* (95% CI) | *β* (95% CI) |
| *Knowledge group* | |  |  |  |  |  |
|  | Quantity of salt consumed | Just the right amount | ref. |  | ref. |  |
|  |  | Too much | 0.6 (-21.7, 22.8) |  | 1.3 (-21.5, 24.2) |  |
|  |  | Too little | 3.1 (-10.3, 16.5) |  | 1.6 (-12.1, 15.4) |  |
|  |  |  |  |  |  |  |
|  | Think dietary salt can cause health problems | No | ref. | ref. | ref. | ref. |
|  |  | Yes | **21.8 (4.1, 39.4)** | **27.1 (7.2, 47.0)** | **21.9 (3.6, 40.2)** | **26.2 (5.7, 46.7)** |
|  |  | Don’t know | 17.2 (-4.7, 39.2) | 19.8 (-5.2, 44.8) | 17.3 (-5.5, 40.1) | 19.2 (-6.7, 45.1) |
|  |  |  |  |  |  |  |
| *Attitude group* | |  |  |  |  |  |
|  | Importance of lowering salt in the diet | Somewhat important | ref. | ref. | ref. | ref. |
|  |  | Very important | -10.5 (-22.9, 1.8) | -12.4 (-25.2, 0.4) | -10.4 (-23.2, 2.3) | -12.2 (-25.4, 1.0) |
|  |  | Not at all important | **37.0 (21.4, 52.5)** | **37.8 (21.4, 54.1)** | **37.6 (21.6, 53.6)** | **38.7 (21.9, 55.6)** |
|  |  |  |  |  |  |  |
|  | Perception of dietary salt | No effect | ref. | ref. | ref. | ref. |
|  |  | Good | **21.3 (5.4, 37.1)** | **25.3 (9.2, 41.5)** | **21.7 (5.3, 38.1)** | **25.7 (9.0, 42.4)** |
|  |  | Bad | 3.6 (-12.4, 19.6) | 8.7 (-7.8, 25.1) | 2.4 (-14.1, 18.8) | 7.4 (-9.5, 24.3) |
|  |  |  |  |  |  |  |
|  | Perception of dietary dawadawa | No effect | ref. |  | ref. |  |
|  |  | Good | -1.9 (-23.9, 20.1) |  | -2.4 (-25.1, 20.2) |  |
|  |  | Bad | No obs. |  | No obs. |  |
|  |  |  |  |  |  |  |
|  | Perception of dietary bouillon | No effect | ref. | ref. | ref. | ref. |
|  |  | Good | -**23.8 (-43.1, -4.5)** | -15.1 (-34.4, 4.1) | **-25.4 (-45.2, -5.5)** | -16.8 (-36.6, 3.0) |
|  |  | Bad | -17.9 (-38.9, 3.1) | -12.3 (-32.9, 8.4) | -15.2 (-37.0, 6.6) | -10.0 (-31.5, 11.5) |
|  |  |  |  |  |  |  |
| *Practice group* | |  |  |  |  |  |
|  | Add salt at the table | Always/often/ sometimes | ref. | ref. | ref. | ref. |
|  |  | Rarely/never | **-16.1 (-26.5, -5.7)** | -5.5 (-17.5, 6.4) | **-15.8 (-26.5, -5.1)** | -5.0 (-17.3, 7.3) |
|  |  |  |  |  |  |  |
|  | Add salt during cooking | Always/often/ sometimes | ref. |  | ref. |  |
|  |  | Rarely/never | 1.3 (-22.9, 25.4) |  | 2.3 (-22.5, 27.0) |  |
|  |  |  |  |  |  |  |
|  | Eat processed foods | Always/often/ sometimes | ref. |  | ref. |  |
|  |  | Rarely/never | 4.4 (-5.6, 14.5) |  | 6.0 (-4.4, 16.3) |  |
|  |  |  |  |  |  |  |
|  | Do at least 1 action regularly to control dietary salt | No | ref. | ref. | ref. | ref. |
|  |  | Yes | **-13.1 (-25.8, -0.4)** | **-19.2 (-36.5, -2.0)** | **-14.6 (-27.6, -1.5)** | **-19.4 (-37.3, -1.6)** |

^1^‘Discretionary salt’ defined as ‘table salt’ and ‘total salt’ defined as discretionary salt plus the proportion of salt from bouillon, which was assumed to be 55% salt (15) . Estimates of Hh discretionary salt and total salt consumption calculated from purchase data using the Fortification Assessment Coverage Toolkit (14). Total households with salt data: n=363; total households with discretionary salt data: n=360. Individual-level factors include data from non-pregnant, non-lactating women of reproductive age (15-49 years, n=239 salt only; n=237 discretionary salt) and non-pregnant lactating women (n=238 salt only; n=235 discretionary salt). KAP predictors were selected *a priori* according to our analysis plan. KAP questions were selected from the World Health Organization (WHO) STEPwise Approach Surveillance (STEPS) Instrument for Noncommunicable Disease Risk Factor Surveillance (version 3.2) (16) and from a KAP questionnaire developed specifically for the pilot survey. The category of KAP attributed to the question was based on the intent of the question (12). CoMIT, Condiment Micronutrient Innovation Trial; Hh, household; KAP, knowledge, attitude, practice; ref., reference group.

^2^ KAP group regression models were linear mixed effects models where each group of predictors that corresponded to Knowledge, Attitudes, or Practices was first tested separately in minimally adjusted model that controlled for Hh size, district (Tolon/Kumbungu), area (urban/rural), and participant type (woman of reproductive age or lactating woman) as fixed effects, and cluster as a random effect. Then, marginally significant KAP predictors (p<0.1, **bolded**) were included in a multivariable model (p<0.5) that controlled for the same variables as the minimally adjusted models.

**Supplemental Table 7.** Estimated per capita discretionary salt consumption (g/d) by asset quintiles and food insecurity status among households participating in the pilot survey

|  | **Asset quintiles** | | | | | | | | | |
| --- | --- | --- | --- | --- | --- | --- | --- | --- | --- | --- |
|  | ***n*** | **1st** | ***n*** | **2nd** | ***n*** | **3rd** | ***n*** | **4th** | ***n*** | **5th** |
| None or Mild FI | *12* | 5.3 (4.7) | *7* | 5.3 (4.1) | *14* | 10.8 (10.4) | *22* | 5.4 (3.9) | *28* | 4.7 (3.0) |
| Moderate FI | *44* | 6.0 (4.6) | *48* | 6.5 (5.7) | *32* | 6.7 (9.5) | *38* | 5.0 (5.5) | *29* | 6.1 (4.9) |
| Severe FI | *18* | 9.2 (8.1) | *19* | 13.2 (15.1) | *25* | 9.6 (7.7) | *16* | 10.2 (9.9) | *11* | 7.2 (7.5) |
| *Total n* | *74* |  | *74* |  | *71* |  | *76* |  | *68* |  |

**Supplemental Table 7 Footnotes:** Per capita discretionary salt consumption presented as mean (SD). Household food insecurity status based on a calculated food insecurity score. 3^rd^ asset quintile and None/Mild Food Insecurity contained an outlier (outlier defined as >60% larger than the value at the 99^th^ percentile; sensitivity analyses examining ANOVA associations between food insecurity or asset quintiles and household discretionary salt consumption with the outlier truncated to the 99^th^ percentile did not change the direction or strength of the associations. FI, food insecurity.

**Supplemental Table 8.** Salt quantity (g) last purchased by asset quintiles among households participating in the pilot survey

|  | **Asset Quintiles** | | | | | |
| --- | --- | --- | --- | --- | --- | --- |
|  | **1^st^** | **2^nd^** | **3^rd^** | **4^th^** | **5^th^** | **Total** |
|  | *n (%)* | *n (%)* | *n (%)* | *n (%)* | *n (%)* | *n (%)* |
| Last purchased 0-299 g salt | 35 (47.3) | 29 (39.2) | 20 (28.2) | 25 (32.9) | 19 (28.0) | 128 (25.3) |
| Last purchased 300-999 g salt | 27 (36.5) | 22 (29.7) | 21 (29.6) | 26 (34.2) | 25 (36.8) | 121 (33.3) |
| Last purchased 1000+ g salt | 12 (16.2) | 23 (31.1) | 30 (42.3) | 25 (32.9) | 24 (35.3) | 114 (31.4) |
| Total | 74 (100) | 74 (100) | 71 (100) | 76 (100) | 68 (100) | 363 (100) |

**Supplemental Table 9.** Characteristics of focus group participants

|  |  | **Total** | **Women 15-49 yrs** | **Women >49 yrs** | **Men** |
| --- | --- | --- | --- | --- | --- |
|  |  | n (%) | n (%) | n (%) | n (%) |
| Total | n | 114 | 56 | 29 | 29 |
| Age | Years | 38 (16,79) | 31 (16,48) | 60 (50, 79) | 35 (20,74) |
| Household size | # household members | 12 (4, 45) | 13 (4, 40) | 12 (4, 36) | 15 (5, 45) |
| Tribe/Group | Mole-Dagbani  Other | 111 (97)  3 (3) | 55 (98)  1 (2) | 29 (100)  0 (0) | 27 (93)  2 (7) |
| Education | No education  Any education  Refused | 82 (72)  31 (27)  1 (1) | 40 (71)  16 (29)  0 (0) | 27 (93)  2 (7)  0 (0) | 15 (52)  13 (45)  1 (3) |
| Marital Status | Single  Married  Divorced/Widowed | 16 (14)  81 (71)  17 (15) | 9 (16)  47 (84)  0 (0) | 0 (0)  12 (41)  17 (59) | 7 (24)  22 (76)  0 (0) |
| Employment Status | Employed/self-employed  Student  Homemaker  Unemployed  Refused | 78 (69)  6 (5)  6 (5)  23 (20)  1 (1) | 35 (63)  4 (7)  5 (9)  12 (21)  0 (0) | 21 (72)  0 (0)  1 (17)  7 (3)  0 (0) | 22 (76)  2 (7)  0 (0)  4 (14)  1 (3) |

^1^Age and household size presented as median (range).

# SUPPLEMENTAL FIGURES

**Supplemental Figure 1.** Distributions of household discretionary salt and total salt consumption (g/d) before and after replacement of values below 2.5 percentile and above 97.5 percentile among participating households in the pilot survey


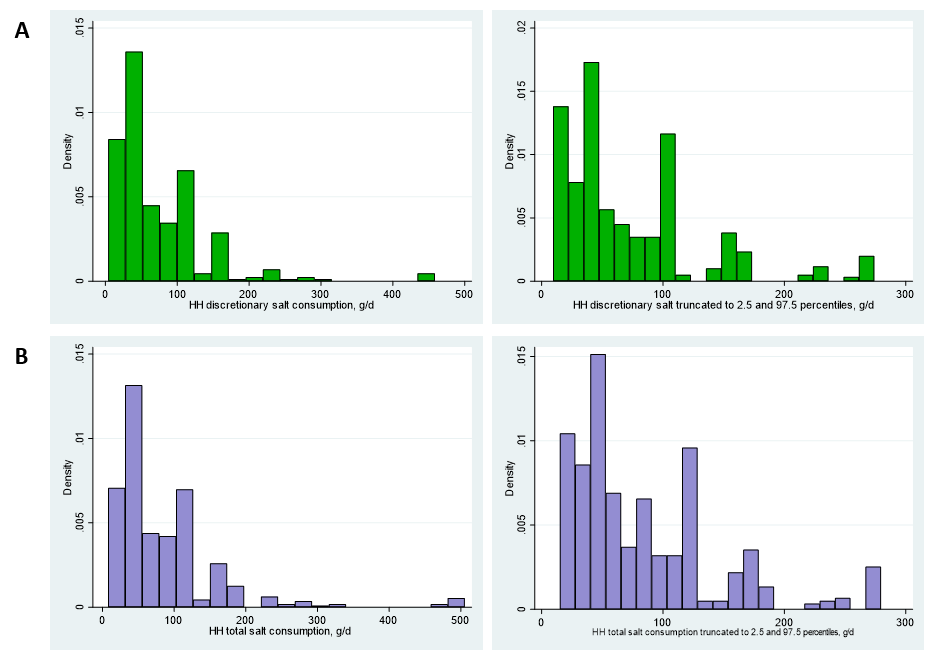


**Legend for Supplemental Figure 1:** Data is from reported household purchase data collected using the Fortification Assessment Coverage Toolkit (14). Values below the 2.5 percentile were replaced with the value at the 2.5 percentile; values above the 97.5 percentile were replaced with the value at the 97.5 percentile. A: Discretionary salt (‘table salt’) distribution with all values (left) and after replacement (right). B: Total salt (discretionary salt + salt from bouillon) distribution with all values (left) and after replacement (right). G/d, grams per day; HH, household; CoMIT, Condiment Micronutrient Innovation Trial.

**Supplemental Figure 2.** Correlations between potential predictors of household salt consumption, including social desirability (SD) variables, among participants in the pilot survey

**Supplemental Figure 2 Legend:** Correlation analysis was conducted in Stata 16 using the ‘polychoric’ command for correlations among ordinal variables. Correlations were considered weak at <0.15 (no color), moderate between 0.2 and 0.4 (orange), or strong at >0.5 (blue). Social desirability (SD) variables were from the Marlowe-Crowne social desirability scale (13). See Supplemental Table 3 for variable definitions. “.” Indicates that a correlation estimate could not be generated due to missing values.

**Supplemental Figure 3.** Flow chart of participating households, participants, and data collected in the pilot survey and focus group discussions


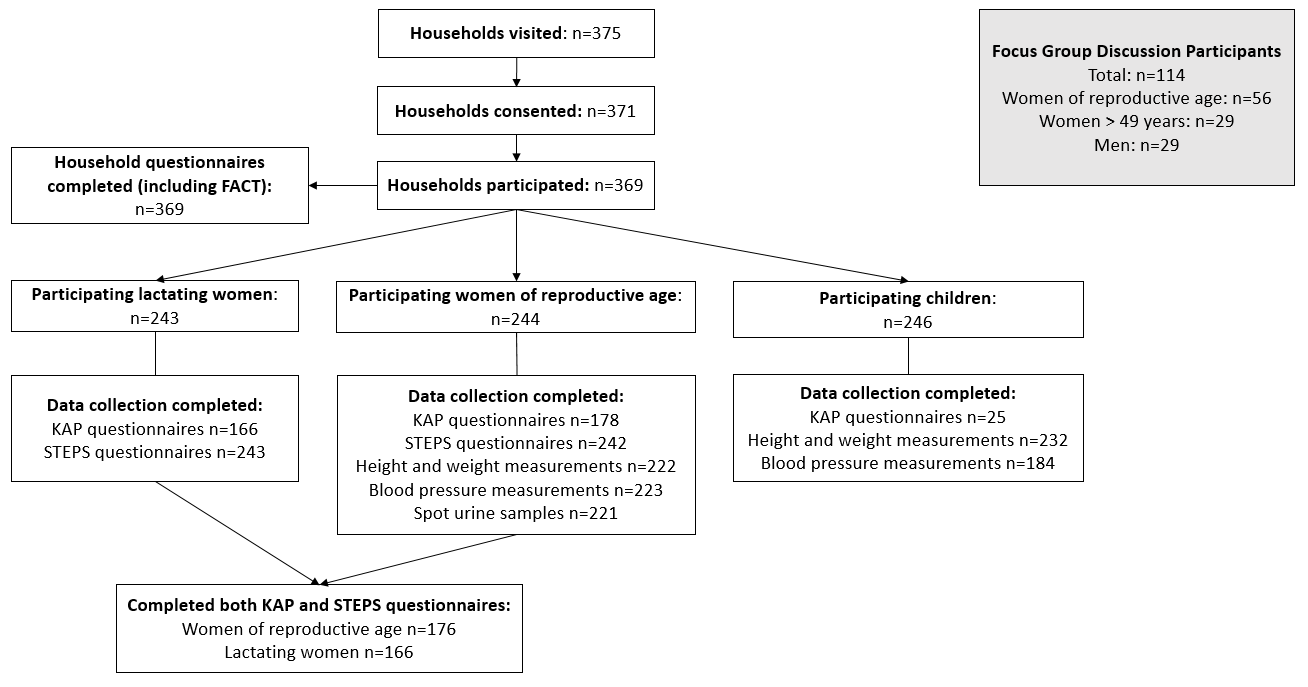


**Legend for Supplemental Figure 3:** In the pilot survey, one KAP questionnaire was completed per household (n=369 total), including n=25 KAP questionnaires completed by caregivers of participating children where the caregiver herself was not a participant. Each participating woman of reproductive age and lactating woman completed the STEPS questionnaire (n=485 total). Abbreviations: FACT, Fortification Assessment Coverage Tool; KAP, Knowledge, Attitudes and Practices; STEPS, WHO noncommunicable disease surveillance tool.

**Supplemental Figure 4.** Factors associated with household salt consumption among women in the pilot survey from mixed effect ANOVA analyses


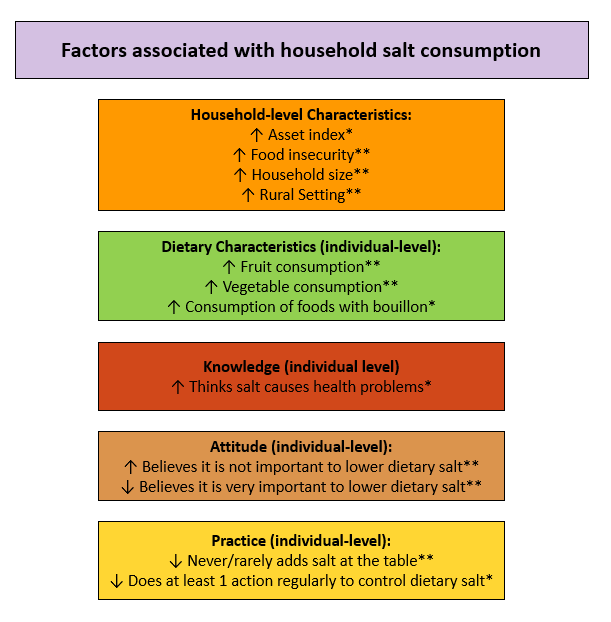


**Supplemental Figure 4 Legend:** ^*^p<0.1; ^**^p<0.05. Factors included are those that were associated with both household discretionary salt (‘table salt’) and total salt (including salt from bouillon) consumption, except “Does at least 1 action regularly to control dietary salt” was only associated in model with household total salt.

# SUPPLEMENTAL APPENDIX

## Focus Group Discussion (FGD) Guide

1. **A household is defined a group of people who recognize the same head of household and who live together and share living expenses and meals. If a man has two or more wives and they and their children live and eat together (even if they eat together only sometimes), they form one household. If the wives and their children do not live together in the same compound and always eat separately, they will form more than one household. Members are included if they have lived with the household at least 6 out of the past 12 months. The following exceptions are always household members: the head of household, any child under 9 months of age, and those who intend to stay in the household for at least 6 months.**
   1. Do you think this definition correctly describes a household? Why or why not?
2. In your home, who usually eats together at meals?
3. How do you determine if someone is not a household member?
4. **Which household member is the main household cook?**
5. If a man has multiple wives, how are cooking duties shared among wives?
6. Who decides what dishes the cook should make in your household?
7. How is food shared with other members of a compound, such as co-wives or other family members not living under the same roof?
8. **Now, let’s talk more specifically about bouillon. Could someone please describe for me what bouillon is?**
   1. How often does your household cook with bouillon?
   2. Does anyone live in a household that does not cook with bouillon?
   3. What are the different types of bouillon products your household cooks with?
   4. With which type of bouillon does your household cook with MOST OFTEN?
   5. Thinking of the type of bouillon you just mentioned, why does your household choose to cook with this type most often?
   6. If this type or brand of bouillon isn’t available, what other types or brands of bouillon would you choose?
   7. During different times of the year, such as during the lean season, do you change how you use bouillon?
   8. Does your household ever use bouillon for purposes other than cooking?
9. **Now let’s talk about bouillon cubes specifically. What are some typical dishes that your household makes with bouillon cubes, that is, dishes your household makes every day or every week?**
   1. Why do you (or the cook of your household) add bouillon cubes to these dishes?
   2. How do you (or the cook of your household) decide how many bouillon cubes to add to these dishes?
   3. Do you always put the entire bouillon cube into the cooking pot or do you save part of it for later use?
   4. If your household doesn’t have any bouillon cubes to add to a dish that calls for bouillon cubes, what do you do?
   5. Why might your household not have any bouillon cubes on hand?
   6. For what other reasons might you not add bouillon cubes to a dish?
10. **When you (or the cook of your household) add bouillon cubes to a dish, how often do you also add salt, dawa dawa, or other seasonings?**
11. When you also add salt, dawa dawa, or other seasonings to a dish made with bouillon, which one is the main flavoring??
12. What dishes do you make every day or every week that use salt, dawa dawa, or other seasonings instead of bouillon?
13. What are some reasons you would add salt, dawa dawa, or other seasonings instead of bouillon to these dish?
14. **How often does your household typically purchase bouillon cubes?**
15. What quantity of bouillon cubes does your household typically purchase at one time?
16. Who in the household decides how often to purchase bouillon?
17. Who in the household decides how much bouillon to purchase at one time?
18. Are the purchased bouillon cubes typically shared between different households on the compound?
19. If your household has less money one week than normal, does your household purchase more, less, or the same amount of bouillon cubes?
20. **What major health problems do you see in this community?**
21. Which, if any, of these health problems is due to a nutritional problem?
22. What do you think could be done to address these health and nutritional problems in your community?
23. **Now we are going to discuss fortified bouillon cubes. What does it mean to have a bouillon cube that is fortified?** *Before continuing with the discussion, assess the group and ensure that each participant understands what ‘fortified’ means.*
24. **What have you heard about fortified bouillon cubes?**
25. If you had to use a fortified bouillon cube in all the dishes where you typically use bouillon cubes, what would be some of your concerns?
26. **If you and your household were consuming dishes made with fortified bouillon cubes, that is bouillon cubes fortified with nutrients like iron, zinc, vitamin A, folic acid, and B12, what, if any, do you think the effects would be on a person’s body?**
27. Who, if anyone, in your household might benefit from consuming fortified bouillon cubes?
28. How might fortified bouillon cubes benefit your or members of your household?
29. Why do you think fortified bouillon cubes might benefit you or your household members?
30. Why might you not want to consume fortified bouillon cubes, or feed dishes with fortified bouillon cubes to your household?
31. **What, if any, impact do you think salt, MSG or dawa dawa has on your body? What impact does it have on the bodies of your children or other household members?**
32. Why do you think these seasonings have these effects on your body and/or that of your children or other household members?

# SUPPLEMENTAL REFERENCES

1. Victora CG, Christian P, Vidaletti LP, Gatica-Domínguez G, Menon P, Black RE. Revisiting maternal and child undernutrition in low-income and middle-income countries: variable progress towards an unfinished agenda. The Lancet. 2021 Apr 10;397(10282):1388–99.

2. World Health Organization. Considerations for quarantine of contacts of COVID-19 cases: Reference number WHO/2019-nCoV/IHR_Quarantine/2021.1 [Internet]. World Health Organization; 2021 [cited 2022 May 29]. Available from: https://www.who.int/publications-detail-redirect/WHO-2019-nCoV-IHR-Quarantine-2021.1

3. World Health Organization Multicentre Growth Reference Study Group. WHO Child Growth Standards: Length/height-for-age, weight-for-age, weight-for-length, weight-for-height and body mass index-for-age: Methods and development. Geneva: World Health Organization; 2006 p. 1–312.

4. Ogedegbe G, Pickering T. Principles and techniques of blood pressure measurement. Cardiol Clin. 2010 Nov;28(4):571–86.

5. Carey RM, Whelton PK, for the 2017 ACC/AHA Hypertension Guideline Writing Committee. Prevention, detection, evaluation, and management of high blood pressure in adults: synopsis of the 2017 American College of Cardiology/American Heart Association hypertension guidelines. Ann Intern Med. 2018 Mar 6;168(5):351.

6. Unger T, Borghi C, Charchar F, Khan NA, Poulter NR, Prabhakaran D, et al. 2020 International Society of Hypertension global hypertension practice guideline. Hypertension. 2020 Jun;75(6):1334–57.

7. World Health Organization. Hypertension Fact Sheet [Internet]. 2021 [cited 2021 Aug 25]. Available from: https://www.who.int/news-room/fact-sheets/detail/hypertension

8. Flynn JT, Kaelber DC, Baker-Smith CM, Blowey D, Carroll AE, Daniels SR, et al. Clinical practice guideline for screening and management of high blood pressure in children and adolescents. Pediatrics [Internet]. 2017 Sep 1 [cited 2021 Aug 25];140(3). Available from: https://pediatrics.aappublications.org/content/140/3/e20171904

9. Imhoff-Kunsch B, Flores R, Dary O, Martorell R. Methods of using household consumption and expenditures survey (HCES) data to estimate the potential nutritional impact of fortified staple foods. Food Nutr Bull. 2012 Sep;33(3 Suppl):S185-189.

10. Brown IJ, Dyer AR, Chan Q, Cogswell ME, Ueshima H, Stamler J, et al. Estimating 24-hour urinary sodium excretion from casual urinary sodium concentrations in western populations: the INTERSALT study. Am J Epidemiol. 2013 Jun 1;177(11):1180–92.

11. Menyanu E, Corso B, Minicuci N, Rocco I, Zandberg L, Baumgartner J, et al. Salt-reduction strategies may compromise salt iodization programs: learnings from South Africa and Ghana. Nutrition. 2021 Apr 1;84:111065.

12. Andrade C, Menon V, Ameen S, Kumar Praharaj S. Designing and Conducting Knowledge, Attitude, and Practice Surveys in Psychiatry: Practical Guidance. Indian J Psychol Med. 2020 Sep 1;42(5):478–81.

13. Crowne DP, Marlowe D. A new scale of social desirability independent of psychopathology. J Consult Psychol. 1960 Aug;24:349–54.

14. Global Alliance for Improved Nutrition (GAIN), Oxford Policy Management. GAIN: Reports and Publications. 2019 [cited 2022 Apr 29]. Fortification assessment coverage toolkit (FACT). Available from: https://www.gainhealth.org/resources/reports-and-publications/fortification-assessment-coverage-toolkit-fact

15. Archer NS, Cochet-Broch M, Mihnea M, Garrido-Bañuelos G, Lopez-Sanchez P, Lundin L, et al. Sodium reduction in bouillon: targeting a food staple to reduce hypertension in sub-Saharan Africa. Front Nutr. 2022 Feb 2;9:746018.

16. World Health Organization. STEPwise Approach to NCD Risk Factor Surveillance (STEPS). [cited 2022 Apr 29]. STEPS Instrument version 3.2. Available from: https://www.who.int/teams/noncommunicable-diseases/surveillance/systems-tools/steps
